# Lymphocyte trajectories after liver transplantation: immune mechanisms, clinical associations, and research priorities

**DOI:** 10.3389/fimmu.2026.1982931

**Published:** 2026-10-19

**Authors:** Jiajun Gao, Zhaoyun Shi, Xuantong Jin, Zhiqi Jiao, Zhenyu Xu, Genhua Ye, Yuhui Wang, Fangliang Guo, Lianbo Yang, Zhenming Gao

**Affiliations:** Division of Hepatobiliary and Pancreatic Surgery, Department of General Surgery, The Second Affiliated Hospital of Dalian Medical University, Dalian, Liaoning, China

**Keywords:** immune monitoring, immune tolerance, infection, liver transplantation, longitudinal trajectories, lymphocyte subsets, rejection, tumour recurrence

## Abstract

Liver transplantation (LT) is an established treatment for end-stage liver disease, yet rejection, infection, biliary injury, post-transplant lymphoproliferative disorder, and tumour recurrence continue to limit outcomes. Post-transplant immune states reflect alloantigen exposure, immunosuppression, microbial or viral stimulation, and inflammation within the graft. This Review examines longitudinal changes in CD8^+^ and CD4^+^ T cells, regulatory and follicular T-cell populations, B cells, natural killer cells, mucosal-associated invariant T cells, and γδ T cells after LT. The most direct evidence concerns acute cellular rejection, in which transient effector activation, T helper 1/interferon-γ responses, multicellular T-cell–B-cell signatures, and graft-local clonal programmes have been described. Early infection has been linked to inhibitory-receptor coexpression and impaired protective function, although treatment exposure, viral history, and phenotype definitions affect interpretation. An increase in circulating regulatory T cells alone does not establish operational tolerance; donor specificity, suppressive stability, and intrahepatic localisation are also required. Evidence relating lymphocyte subsets to biliary, vascular, metabolic, and oncological outcomes is less direct and often comes from experimental or non-transplant studies. Meaningful interpretation requires attention to sampling time, anatomical compartment, cellular function, and treatment exposure. Serial blood phenotyping may be combined with functional assays, soluble immune-mediator or protein signatures, graft histology, immune-receptor repertoires, and selected single-cell or spatial analyses. This approach defines research priorities, not a validated score or treatment algorithm. Clinical use will require standardised sampling, prespecified outcomes, externally validated thresholds, and prospective strategy trials that assess both benefit and harm.

## 1 Introduction

Advances in immunosuppression and perioperative care have improved survival after liver transplantation (LT), but post-transplant complications still affect long-term outcomes. Rejection remains clinically relevant, whereas prolonged immunosuppression increases susceptibility to opportunistic infection, malignancy, and metabolic disease. Biliary complications and ischaemia–reperfusion injury (IRI) may further compromise graft function. Research has consequently moved beyond the overall intensity of immunosuppression to measurable differences in immune-subset composition and function. Current areas of interest include T-cell differentiation and exhaustion, regulatory T-cell (Treg) abundance and localisation, T follicular helper (Tfh)–B-cell cooperation, and changes in innate-like lymphocytes such as mucosal-associated invariant T (MAIT) cells (1–5). We examine how these populations change over time, how those changes relate to complications, and which observations may be clinically useful. Graft-local findings are considered alongside peripheral blood measurements because blood does not consistently represent immune activity within the allograft (3, 4).

Post-transplant morbidity spans immune, infectious, structural, metabolic, and oncological conditions. Recipients may develop T cell-mediated rejection (TCMR), antibody-mediated rejection (ABMR), cytomegalovirus (CMV) or Epstein–Barr virus (EBV) reactivation, biliary or vascular disease, metabolic syndrome, or post-transplant diabetes mellitus (PTDM). *De novo* malignancy, post-transplant lymphoproliferative disorder (PTLD), and hepatocellular carcinoma (HCC) recurrence present additional risks. These events arise from different biological and treatment-related factors, occur at different times, and may require opposing changes in immunosuppression. A single immune marker rarely provides reliable prospective discrimination across these settings.

The liver has immunological features that distinguish it from kidney and heart grafts. Portal blood continuously delivers gut-derived antigens and pathogen-associated molecular patterns (PAMPs). Kupffer cells, liver sinusoidal endothelial cells, and intrahepatic antigen-presenting cells can maintain local immune restraint while retaining the capacity for rapid inflammatory responses. After LT, immunosuppression does not produce uniformly reduced responsiveness. The observed immune state varies with persistent antigen exposure, IRI, changes in the microbial ecosystem, and treatment.

The populations considered here include CD8^+^ and CD4^+^ T cells, Tregs, Tfh cells, T follicular regulatory (Tfr) cells, B cells, and natural killer (NK) cells, together with innate-like MAIT and γδ T cells. They contribute to alloimmune injury, antimicrobial defence, and tumour surveillance. Cell abundance captures only one aspect of these responses. Differentiation, cytotoxicity, cytokine production, metabolic adaptation, and distribution between blood and graft also influence clinical interpretation. Longitudinal measurements may detect shifts towards rejection, infection susceptibility, or tolerance that are missed by a single time point.

The Review is organised around time, anatomical compartment, and cellular function (**Figure 1**). We first consider the immune exposures and sampling conditions that alter marker interpretation. Subsequent sections examine individual lymphocyte populations and direct clinical associations with rejection, infection, tolerance, tumour outcomes, and other complications. The final sections address monitoring, validation, and intervention studies. **Figure 1** is an organising model for research evidence and is not a validated risk score or treatment algorithm.

**Figure 1:**
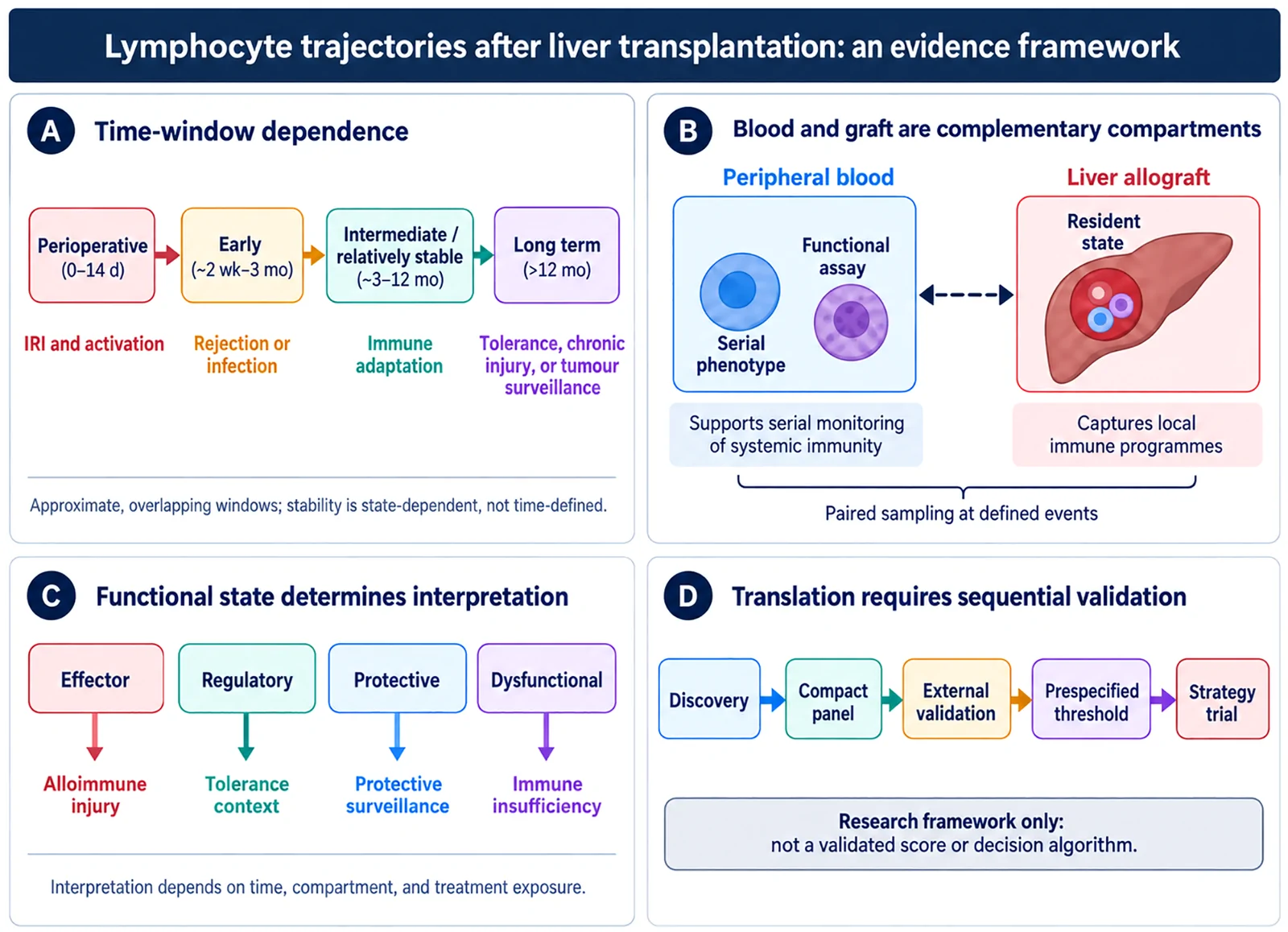
Evidence framework for interpreting lymphocyte trajectories after liver transplantation. **(A)** Post-transplant immune signals are interpreted across approximate and overlapping perioperative (transplantation to ~14 days), early (~2 weeks–3 months), intermediate/relatively stable (~3–12 months), and long-term (>12 months) windows; clinical stability is state-dependent rather than defined by elapsed time alone. **(B)** Peripheral blood supports serial phenotyping and functional testing, whereas the liver allograft captures resident and graft-local programmes; targeted or event-driven paired sampling is most informative when tissue diagnosis or local mechanistic assessment is required. **(C)** Effector, regulatory, protective, and dysfunctional states can have different implications for alloimmune injury, tolerance, immune surveillance, and immune insufficiency. **(D)** Translation requires sequential progression from discovery to a compact panel, external validation, prespecified thresholds, and a strategy trial. The framework organises research evidence and is not a validated risk score or treatment algorithm. IRI, ischaemia–reperfusion injury.

## 2 Methods

### 2.1 Review design and scope

This structured narrative review integrates clinical, tissue-based, and mechanistic evidence on lymphocyte remodelling after LT. It does not claim exhaustive coverage, provide pooled effect estimates, or meet the requirements of a systematic review. No protocol was registered because the initial project corpus had been assembled through several complementary retrieval routes rather than a single prospectively defined search.

### 2.2 Evidence identification and eligibility

Candidate publications were identified from a project evidence matrix linked to the available full texts and reference records. The initial corpus covered rejection, infection, operational tolerance, IRI, biliary complications, tumour outcomes, lymphocyte-directed interventions, and immune monitoring after LT. Because it did not originate from one registered query, the corpus was treated as a structured source base rather than as the yield of a reproducible systematic search.

Literature identification focused primarily on studies published from 2015 to 2026. Titles and abstracts were screened for relevance, and full texts were reviewed when available. Earlier publications were retained only when they provided essential mechanistic or historical context. Bibliographic fields were checked against PubMed for indexed records.

Eligible primary studies examined a defined lymphocyte population in LT recipients, human liver-allograft tissue, or an LT model and reported a transplant outcome or mechanistic endpoint. Reviews and consensus documents were used for biological, diagnostic, or reporting context, not as primary evidence for clinical associations. Evidence from other transplanted organs, graft-versus-host disease, or non-transplant liver disease was retained only when it addressed an unresolved LT pathway. Such evidence was labelled as indirect and was not presented as an established LT mechanism. Duplicate records, non-independent supplementary reports, and publications without a defensible link to LT complications or lymphocyte biology were excluded.

### 2.3 Evidence extraction and synthesis

For each publication, we recorded the study setting, population or model, lymphocyte definition, sample compartment, sampling time, design, analytical method, comparator, outcome, direction of association, and major limitations. Results reported by the original investigators were separated from their interpretation and from the synthesis presented here. Quantitative thresholds, intervention effects, and mechanistic claims were retained only when they could be traced to the cited source.

Evidence was grouped into three levels. Direct evidence comprised studies in LT recipients or human liver-allograft tissue with a defined transplant outcome. Mechanistic evidence included functional assays, perturbation experiments, histology, single-cell or spatial profiling, and immune-receptor analyses in LT or closely related settings. Indirect evidence came from other-organ transplantation, graft-versus-host disease, or non-transplant liver disease. **Table 1** summarises the principal direct human LT studies used for clinical interpretation.

**Table 1:** Direct human liver-transplant evidence linking lymphocyte measurements to post-transplant outcomes.

Evidence domain	Study design and population	Immune readout and compartment	Reported association or result	Main limitation/translational status
Acute rejection (1)	Case–control study in LT recipients	Peripheral IFN-γ^+^CD4^+^ T cells and GZMB^+^CD19^+^ B cells	Both populations increased during ACR; their combination improved discrimination.	No validated independent threshold; biopsy remains necessary.
Early infection (2)	Prospective peri-transplant cohort	Peripheral PD-1^+^TIM-3^+^ memory T cells and IFN-γ function	Higher inhibitory-receptor coexpression and lower IFN-γ production were associated with infection within 90 days.	Phenotype was not CD8-specific; no multicentre threshold.
Treg expansion (3)	Low-dose IL-2 study in stable LT recipients	Circulating and graft Tregs; donor reactivity	Circulating Tregs expanded without donor-specific enrichment or consistent graft homing; tolerance was not established.	Small interventional cohort; expansion alone is not a tolerance marker.
Blood–graft concordance (4)	Paired blood and liver-allograft TCR repertoire study	TCR clonotypes in blood and graft	Repertoires differed substantially, with greater convergence during rejection.	Tissue access and event-driven sampling limit routine use.
MAIT remodelling (5)	Cross-sectional comparison of tolerant and immunosuppressed LT recipients	Peripheral MAIT abundance and function	Persistent numerical and functional impairment differed by clinical state.	No prospective outcome threshold.
Rejection tissue programmes (18–22)	Human biopsy single-cell, spatial, and multi-tissue studies	Clonally expanded, resident, cytotoxic, interferon, and JAK–STAT immune states	Defined multicellular graft programmes associated with T-cell-mediated rejection.	External validation and clinically deployable assays are needed.
Perioperative IRI (26)	Human comparison of ischaemia-free and conventional LT	Graft CD69^+^CD103^-^CD8^+^ T cells and HIF-2α-related state	Reduced ischaemic injury accompanied lower accumulation of the implicated CD8^+^ state.	Specific perioperative setting; later complication prediction untested.
Th17/Treg balance (27, 29)	Serial peripheral immune phenotyping in LT cohorts	Th17/Treg ratio and early Treg change	Higher Th17/Treg imbalance or early Treg decline accompanied rejection-related outcomes.	Sampling and treatment protocols differed; proposed time-point thresholds are not generalisable.
Tfh17, IRI, and DSA (35)	Prospective pretransplant cohort	Circulating Tfh17 and donor-reactive Tfh cells	Pretransplant Tfh17-related states were associated with IRI and subsequent DSA development.	Single-cohort association requiring replication.
Operational tolerance (46–48, 57)	Human blood, graft, and donor-reactivity studies	Donor-reactive Tregs, hepatic localisation, and senescence-associated states	Tolerance-associated regulation depended on specificity, localisation, and cellular state.	No single marker or validated withdrawal rule.
Regulatory-cell therapy (49, 50)	Pilot and early-phase living-donor LT studies	Infused regulatory-cell products and immune monitoring	Treatment was feasible in selected recipients and supported immunosuppression reduction in some participants.	Small, selected, intensively monitored cohorts; routine efficacy unproven.
HCC recurrence (62)	Clinical and mechanistic LT cohort	CXCL10/CXCR3-dependent Treg recruitment to the graft	Graft injury-associated Treg recruitment was linked to HCC recurrence.	Association is not a validated recurrence score.
NK-associated outcomes (67, 68)	Paediatric viral co-infection cohort and adult HCC cohort	Peripheral NK phenotype or pretransplant NK activity	Altered NK states accompanied PTLD in co-infected children; lower NK activity was associated with HCC recurrence.	Small, outcome-specific cohorts; findings are not interchangeable.
Infection monitoring (75–77)	Prospective ICU and paediatric LT cohorts	Lymphocyte count/function, CD3^+^ lymphopenia, and EBV-specific T cells	Results were mixed: one study favoured monocyte HLA-DR, while paediatric studies linked CD3^+^ lymphopenia or weak EBV-specific responses to infection.	Assays, ages, pathogens, and endpoints differed; thresholds are not transferable.
MAIT recovery and rejection (70, 71)	Longitudinal human cohort and human/mouse multi-omic study	Peripheral MAIT recovery and MAIT-associated graft programmes	MAIT-cell trajectories accompanied clinical recovery and rejection-related immune states.	Prospective outcome-specific validation remains necessary.
Complementary soluble/cellular signature (96)	Prospective multicentre paediatric LT cohort	Plasma soluble immune mediators with peripheral cellular phenotyping	One favourable multivariable plasma signature emerging from approximately 2 weeks was associated with rejection-free survival during the first year and a higher CD56^bright^ NK-cell frequency.	Paediatric cohort; stability, thresholds, and transferability to adults require external validation.

The table prioritises studies in recipients or human liver-allograft tissue with defined transplant outcomes and does not include all indirect or experimental evidence discussed in the text. ACR, acute cellular rejection; DSA, donor-specific antibody; HCC, hepatocellular carcinoma; IRI, ischaemia–reperfusion injury; LT, liver transplantation; MAIT, mucosal-associated invariant T; NK, natural killer; PTLD, post-transplant lymphoproliferative disorder; TCR, T-cell receptor; Treg, regulatory T cell.

Observational associations were interpreted as non-causal. We did not apply a formal risk-of-bias instrument or certainty-grading system. Greater interpretive weight was given to longitudinal human LT studies, graft-level measurements, functional validation, clinically defined outcomes, and independent replication. Differences were assessed in relation to sampling time, biological compartment, phenotype definition, treatment exposure, and outcome classification. Proposed monitoring panels and artificial intelligence applications are research proposals rather than validated diagnostic or treatment-selection tools.

### 2.4 Use of generative artificial intelligence

OpenAI Codex version 26.818.8289.0, powered by the GPT-5.6 model (OpenAI, San Francisco, CA, USA), assisted with literature organisation, English-language editing, schematic figure preparation, and document formatting. The authors checked every citation, factual statement, interpretation, and figure against the cited sources and approved the final manuscript. The tool was neither an author nor an evidence source.

## 3 Post-transplant complications and lymphocyte remodelling

After transplantation, persistent alloantigen exposure, uneven suppression of immune subsets, microbial or viral reactivation, and inflammation-repair coupling keep the immune system out of equilibrium. Four observations recur across the literature. Abundance and function often diverge: low-dose interleukin-2 (IL-2) expands circulating Tregs without enriching donor-specific cells or promoting their homing to the liver allograft. The same intervention can induce intrahepatic interferon-γ (IFN-γ)-associated inflammation and hinder immunosuppression withdrawal (3).

Sampling time can change the meaning of an immune marker. Increased coexpression of programmed cell death protein 1 (PD-1) and T-cell immunoglobulin and mucin-domain-containing 3 (TIM-3) on memory T cells before or soon after transplantation has been linked to infection within 90 postoperative days and to reduced IFN-γ production *in vitro* (2). Anatomical location is equally relevant: T-cell receptor (TCR) repertoires in the graft differ substantially from those in peripheral blood, and donor-reactive CD4^+^ T-cell and Treg clones can be enriched in the liver (4). Tregs, regulatory B cells (Bregs), MAIT cells, and γδ T cells have also been implicated in IRI, biliary inflammation, and chronic immune remodelling, but much of this evidence is experimental or indirect (5–8). Interpretation must account for the immune stimulus, time window, sample compartment, and co-occurring clinical events.

### 3.1 Three drivers of the post-transplant immune milieu: alloimmunity, immunosuppression, and persistent antigen or microbial exposure

Three forces act on the recipient immune system at the same time. Donor antigens elicit alloimmune responses; immunosuppression weakens protective immunity and permits opportunistic pathogens to reactivate; surgery-related IRI and changes in the gut–liver axis sustain inflammation–repair coupling. Because these forces coexist, one pathway can have opposing associations with different outcomes. Inhibitory-receptor and exhaustion phenotypes, for example, may lower rejection risk while increasing susceptibility to infection (2, 9). The following subsections examine how these overlapping drivers vary across time and anatomical space.

### 3.2 Time-window specificity: from perioperative inflammation to long-term immune remodelling

For interpretive purposes, the perioperative period can be considered from transplantation to approximately postoperative day 14, the early period from about 2 weeks to 3 months, an intermediate or relatively stable period from approximately 3 to 12 months, and the long-term period beyond 12 months. These intervals are approximate and overlapping rather than validated biological stages or fixed clinical cut-offs. Innate inflammation and rapid T-cell activation soon after surgery have been linked to acute rejection, whereas continued antigenic stimulation, immunosuppression, viral exposure, and immune reconstitution progressively alter phenotype and function thereafter. CMV exposure and immunosuppressive drugs can confound peripheral tolerance-associated signatures (10), whereas inhibitory-receptor expression has been associated with infection risk (2). Importantly, a relatively stable post-transplant state should not be defined by elapsed time alone; it can be operationally characterised by sustained graft biochemical stability, absence of recent rejection or active infection, relatively stable immunosuppressive exposure, and no major recent therapeutic change. A measurement taken at one time point must therefore be interpreted within both its approximate sampling window and the recipient’s contemporaneous clinical state.

### 3.3 Spatial compartmentalization: peripheral immune markers cannot fully represent the graft-local immune state

Tissue studies increasingly show why sampling location changes interpretation. TCR repertoire analyses find donor-reactive clones enriched in the liver allograft and document marked differences from peripheral blood. Only during strong immune events, such as acute rejection, do peripheral and intrahepatic repertoires become more similar (4). Peripheral blood therefore offers low-risk repeatability and high temporal resolution for longitudinal trends, whereas graft tissue provides local histological and spatial resolution. These compartments are complementary rather than interchangeable. When rejection or another graft-local process is suspected, tissue assessment remains important, but repeated biopsy is constrained by invasiveness, procedural risk, sampling error, spatial heterogeneity, and the selection and timing bias inherent in clinically indicated sampling (4, 37, 73). Across the approximate post-transplant windows defined above, serial peripheral sampling is particularly suitable for capturing rapid perioperative and early immune changes, whereas graft tissue becomes most informative when graft dysfunction, suspected TCMR or ABMR, or discordant circulating signals emerge. During relatively stable or long-term follow-up, tissue sampling is generally reserved for defined clinical indications, protocolised tolerance or immunosuppression-withdrawal studies, or unresolved graft abnormalities, depending on centre-specific practice.

Less-invasive circulating approaches can complement both compartments. Multiplex soluble immune-mediator or protein signatures may provide an integrated systemic readout, while donor-derived cell-free DNA can reflect graft injury before or during dysfunction; neither is lymphocyte specific, and neither should replace histology when tissue diagnosis is clinically required (96, 97).

Cellular origin adds a further layer to graft interpretation. HLA-mismatched human allografts show rapid recipient leukocyte infiltration and progressive recipient-cell replenishment of several graft immune compartments, while smaller donor-derived pools of tissue-resident memory T cells and Kupffer cells can persist for more than a decade; recipient-derived cells can also acquire liver-resident phenotypes (91). Long-lived donor-derived mononuclear phagocytes, including Kupffer cells with antigen-presenting capacity, further indicate that donor-origin immune persistence is not restricted to lymphocytes. Donor-derived liver-resident NK cells can also persist for years, and donor NK and CD56^+^ T-cell populations may transiently enter the recipient circulation after transplantation (92, 93). Thus, donor-derived is not synonymous with tissue-resident, and recipient-derived is not synonymous with circulating. HLA mismatch and other genotype-based chimerism approaches can help distinguish origin, but low abundance, sampling limitations, and phenotypic convergence restrict interpretation. Current evidence does not establish that donor-cell persistence itself denotes tolerance or that recipient-cell replacement denotes rejection, and whether these origin dynamics independently correlate with tolerance, rejection, infection, or long-term outcome remains insufficiently defined.

### 3.4 Recurrent signals and their evidentiary limits

Several signals recur across distinct evidence streams, although their directness varies. The balance between effector and regulatory activity is more informative than the abundance of a single subset; Treg expansion, for instance, may not confer tolerance without donor specificity and hepatic homing (3, 11). Exhaustion and inhibitory-receptor pathways have context-dependent effects after LT. PD-1/PD-L1 signalling may help maintain an immunosuppressive milieu through plasmacytoid dendritic cell (pDC)-associated mechanisms (9), yet the same axis is linked to infection risk and impaired function (2). Multicellular signatures also perform better than isolated markers. Combined IFN-γ^+^CD4^+^ T-cell and granzyme B-positive (GZMB^+^) CD19^+^ B-cell measurements discriminate acute rejection more effectively than either population alone (1). Function may be closer to the underlying complication mechanism than phenotype: reduced MAIT-cell abundance and weak responses to bacterial stimulation suggest impaired protective immunity (5), whereas IL-15-driven bypass activation can restore cytotoxicity during inflammation (12). The next subsection considers how these patterns interact when complications occur together.

### 3.5 A systems-immunology view of co-occurring complications

Treatment decisions expose the close relationship between post-transplant complications. Intensifying immunosuppression to control rejection increases infection risk; reducing treatment during infection can precipitate rejection. Shared immune pathways add to these treatment effects. Exhaustion-associated phenotypes have been linked to infection (2), whereas inhibitory pathways may contribute to a tolerogenic milieu (9). The same pathway may protect the graft while weakening antimicrobial defence. Management must balance these competing risks for the individual recipient.

## 4 Lymphocyte modules linking immune state to complications

Lymphocyte remodelling after liver transplantation comprises state transitions rather than a linear shift from activation to suppression. Alloantigen exposure, IRI, microbial signals, immunosuppressive therapy, and the graft microenvironment shape these transitions. The meaning of a cell state depends on when it appears, where it is measured, and whether its effector or regulatory programme is functional. A transient effector response may participate in early graft injury; persistence of the same phenotype may instead reflect tissue residency, chronic antigen exposure, or incomplete resolution. Numerical recovery of a regulatory or antimicrobial subset likewise does not establish restored donor specificity, suppressive capacity, or protective immunity.

The evidence base supports three levels of interpretation. Mechanistic evidence derives from perturbation experiments, animal models, functional assays, and single-cell or immune-repertoire analyses. Clinical evidence consists mainly of associations between immune phenotypes and defined post-transplant outcomes. Proposed trajectory models integrate these observations into testable hypotheses but do not establish causality. This distinction is essential because immunosuppressive exposure, infection, sampling time, and the marked differences between blood and graft compartments can each alter the observed phenotype.

### 4.1 CD8^+^ T cells: effector, memory, resident, and exhaustion states across rejection and infection

CD8^+^ T cells can injure the allograft while also maintaining antiviral and antitumour surveillance. Their post-transplant course is better described by the balance among cytotoxic effector, effector-memory, tissue-resident memory, and dysfunction-or exhaustion-associated states than by absolute abundance alone. Alloimmune activation begins when recipient CD8^+^ T cells recognise donor antigens. Direct recognition of intact donor peptide–major histocompatibility complex (MHC) class I complexes can initiate a strong early response, whereas recipient antigen-presenting cells can sustain CD8^+^ activation through cross-presentation of donor-derived peptides. TCR signalling, costimulation, and inflammatory cytokines promote clonal expansion and cytotoxic differentiation. Activated cells release IFN-γ, granzyme B, and perforin and can engage death-receptor pathways, thereby contributing to hepatocyte, cholangiocyte, and endothelial injury during T cell-mediated rejection (13, 14). The relative contribution of these pathways in human liver transplantation remains difficult to resolve because most clinical studies are observational.

Memory differentiation can prolong the response beyond the initial alloimmune episode. Effector-memory CD8^+^ T cells respond rapidly to renewed antigen exposure, whereas tissue-resident memory T cells (TRM) remain in the graft and can react locally without recirculating through blood. Single-cell RNA sequencing of human and experimental liver allografts identified CD8^+^ TRM populations enriched in rejection-associated tissue and linked their persistence to sustained intrahepatic alloimmunity (15). In murine liver and heart transplantation, donor tissue-resident lymphocytes persisted after MHC-matched grafting, whereas MHC mismatch promoted recipient-cell infiltration and loss of donor lymphocytes (16). Human HLA-mismatched allograft studies provide complementary evidence: small donor-derived pools of TRM and Kupffer cells can persist for more than a decade despite rapid recipient-cell infiltration, and recipient-derived T cells can acquire CD69/CD103 tissue-resident features within the graft (91). These findings show that cellular origin and tissue residency are related but non-equivalent dimensions of graft immunity. Reviews of hepatic TRM biology provide phenotypic and functional context but do not establish post-transplant outcomes (17). These observations are consistent with a graft-resident rejection programme, although enrichment in rejecting tissue does not show that TRM cells initiated the event. Human biopsy studies have extended this model. Expanded intragraft CD8^+^ clonotypes persisted after histological resolution of TCMR and retained treatment-resistant effector or NK-like programmes. Combined single-cell and spatial transcriptomics also identified CD4^+^ effector-like and CD8^+^ precursor-exhausted or transitional-effector states concentrated in rejecting grafts (18, 19).

Spatial studies further illustrate the difference between blood and graft. Imaging mass cytometry of liver-allograft biopsies identified cellular neighbourhoods containing proliferating and cytotoxic CD8^+^ T cells, PD-1^+^ T cells, Tregs, and polarised macrophages during active TCMR (20). The coexistence of effector, regulatory, and inhibitory phenotypes points to a multicellular tissue programme rather than expansion of one circulating subset. Multi-tissue profiling of 98 paediatric recipients identified HLA-DR-high and PD-1-high T-and B-cell states during TCMR and a tissue-correlative 11-protein plasma panel, which still requires external validation (21). In 13 adult recipients, longitudinal multimodal profiling detected a time-dependent JAK–STAT–IFN programme. Early post-reperfusion activity characterised permissive grafts, whereas later activity marked subclinical rejection; ruxolitinib suppressed alloreactive CD8^+^ T-cell proliferation *in vitro* (22).

Persistent alloantigen or viral stimulation can induce an exhaustion-associated state marked by PD-1, TIM-3, lymphocyte-activation gene 3 (LAG-3), and impaired cytokine or cytotoxic responses. Direct liver-transplant evidence is strongest for PD-1 and TIM-3. A high pretransplant frequency of PD-1^+^TIM-3^+^ memory T cells was associated with subsequent infection, and these cells produced less IFN-γ ex vivo (2). The study was not CD8-specific, and comparable clinical evidence for LAG-3 remains limited. PD-1^+^ CD8^+^ T cells are also enriched within rejecting grafts (20), showing that inhibitory-receptor expression can coexist with active inflammation and should not be equated with terminal dysfunction.

The clinical consequence of this state depends on the dominant immune pressure. Persistent functional impairment may weaken control of CMV, EBV, and other pathogens, whereas adaptive inhibitory signalling within the graft may restrain excessive alloimmune injury. Distinguishing these possibilities requires functional measurements, including proliferation, cytokine production, and cytotoxicity, rather than receptor expression alone. TCR repertoire analysis provides clonal evidence that circulating CD8^+^ phenotypes do not fully represent intrahepatic immunity. Repertoires in liver allografts differ substantially from those in peripheral blood (4). A graft-enriched clone may reflect local antigen recognition, selective recruitment, or tissue retention, whereas its absence from blood does not indicate immunological quiescence. Longitudinal paired blood-biopsy studies are needed to determine whether specific clonotypes precede rejection, persist after treatment, or accompany stable graft accommodation.

Peripheral hyporesponsiveness is also context specific. Beyond the first year, simultaneous liver-kidney recipients had lower circulating CD8^+^, activated CD4^+^, and effector-memory T-cell frequencies and weaker donor-specific proliferation and IFN-γ responses than kidney-alone recipients (23). Their profiles resembled those of solitary liver recipients. In a prospective living-donor LT study, donor regulatory dendritic-cell infusion before transplantation reduced T-bet-high Eomes-high memory CD8^+^ T cells and increased the Treg-to-effector-memory CD8^+^ ratio (24). These studies link donor-specific function and treatment exposure to circulating phenotypes but do not define outcome thresholds.

CD8^+^ T-cell monitoring requires both temporal and spatial information. Peripheral activation and exhaustion-associated phenotypes may inform risk assessment, but they cannot replace graft evaluation when rejection is suspected. In an experimental model, hepatic expression of an allogeneic MHC class I molecule generated alloantigen-specific suppressive CD8^+^ T cells and prolonged pancreatic islet-allograft survival (25). This liver-directed tolerance model is consistent with regulatory CD8^+^ states but does not establish their role in human LT. Research endpoints could combine effector-memory and TRM states, functional exhaustion, viral burden, and paired TCR repertoires. No validated CD8-specific threshold distinguishes rejection, infection susceptibility, and adaptive immune restraint across clinical settings. **Figure 2** summarises this state-and compartment-dependent interpretation. Paired clinical and experimental analyses also linked post-reperfusion HIF-2α activity in CD69^+^CD103^-^CD8^+^ T cells to graft injury after conventional LT; ischaemia-free LT and experimental HIF-2α inhibition attenuated the same programme (26).

**Figure 2:**
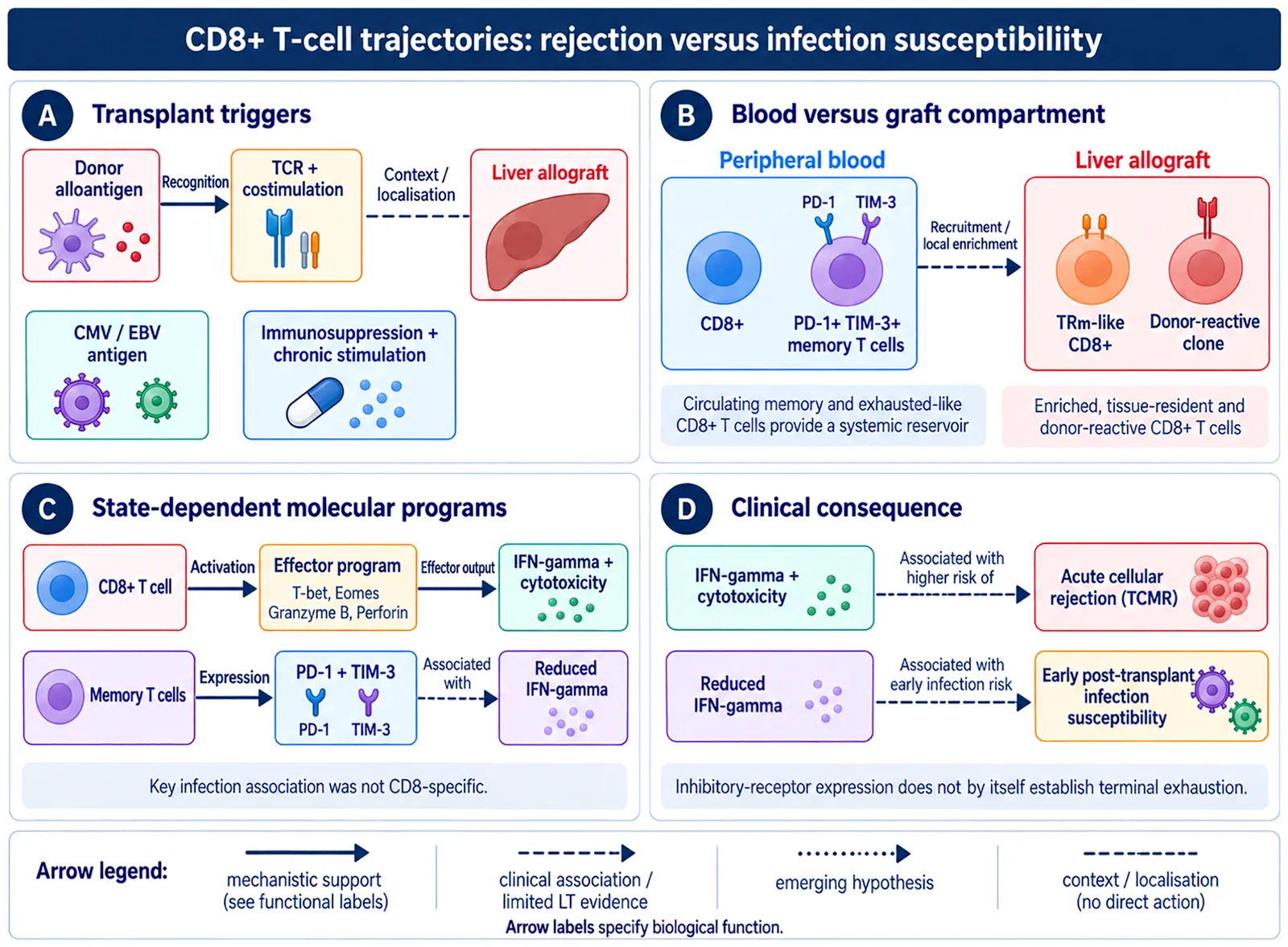
CD8^+^ T-cell trajectories across rejection and infection susceptibility. **(A)** Transplant-related triggers. **(B)** Cell states in peripheral blood and the liver allograft. **(C)** Effector programmes and the association between inhibitory-receptor expression and reduced interferon-γ production. **(D)** Associations with rejection and early infection susceptibility. Donor alloantigen, viral stimulation, immunosuppression, and graft localisation shape the balance between cytotoxic effector, tissue-resident, and exhaustion-associated states. Recruitment and local enrichment connect circulating and graft-local populations. Graft-enriched clones and effector programmes may accompany T cell-mediated rejection, whereas PD-1^+^TIM-3^+^ memory phenotypes with reduced interferon-γ production are associated with early infection risk. The reported early-infection association was not CD8^+^ T-cell specific and does not establish CMV- or EBV-specific risk. Inhibitory-receptor expression alone does not establish terminal exhaustion. CMV, cytomegalovirus; EBV, Epstein–Barr virus; MHC, major histocompatibility complex; PD-1, programmed cell death protein 1; TCR, T-cell receptor; TCMR, T cell-mediated rejection; TIM-3, T-cell immunoglobulin and mucin-domain-containing 3; TRM, tissue-resident memory T cell.

### 4.2 CD4^+^ T cells: Th1/Th17 responses and Tfh/Tfr–B-cell cooperation

CD4^+^ T cells coordinate alloimmune inflammation, humoral immunity, and regulation through functionally distinct but plastic states. Antigen presentation, costimulation, local cytokines, and immunosuppressive exposure can favour Th1, Th17, T follicular helper (Tfh), or regulatory programmes. Their clinical effects emerge through interactions with macrophages, neutrophils, B cells, and Tregs rather than through isolated changes in CD4^+^ T-cell abundance.

Th1 differentiation links donor-antigen recognition to macrophage-rich graft inflammation. Activated Th1 cells secrete IFN-γ, which enhances antigen presentation, activates macrophages, and promotes inflammatory chemokine production. These changes can recruit additional effector lymphocytes into portal and perivascular regions and amplify T cell-mediated injury. The pathway is mechanistically plausible and consistent with the inflammatory architecture of acute rejection, although human studies have not quantified the independent contribution of Th1 cells to graft damage (14).

Clinical evidence supports a coordinated Th1-B-cell signature. In a case-control study, increased percentages of IFN-γ^+^CD4^+^ T cells and granzyme B^+^CD19^+^ B cells were jointly associated with acute hepatic rejection (1). This combined phenotype may capture broader effector activation, but the study did not demonstrate direct T–B-cell cooperation or establish either population as an independent predictor.

Th17 cells provide another inflammatory route through interleukin-17 (IL-17)-dependent recruitment of neutrophils and inflammatory monocytes. Serial studies have linked changes in the Th17/Treg balance to rejection-related states and immunosuppression tapering after liver transplantation (27–29). This ratio is a candidate monitoring measure, but its direction and magnitude vary with sampling time, immunosuppressive regimen, and endpoint definition.

The proposed link between Th17 cells and biliary injury requires stricter evidentiary boundaries. Experimental autoimmune cholangitis, biliary atresia, and cholestatic liver-disease models show that Th1/Th17 or IL-17 pathways can promote cholangiocyte-associated chemokine production and inflammatory-cell recruitment (7, 30–33). These studies establish an IL-17-centred mechanism in non-transplant biliary inflammation. They do not prove that Th17 cells cause post-transplant biliary strictures, ischaemic cholangiopathy, or other surgically and ischaemically mediated biliary complications.

Tfh cells provide cognate help to antigen-activated B cells, promoting germinal-centre selection, class switching, plasmablast differentiation, and donor-specific antibody production. Tfr cells can restrain this response by limiting Tfh activity and B-cell selection. In primary biliary cholangitis, pyruvate dehydrogenase complex E2 (PDC-E2)-reactive conventional and regulatory CD4^+^ clones occur with a Tfh-skewed conventional-cell phenotype, providing disease-specific evidence for antigen-targeted regulation (34). This non-transplant observation does not establish a mechanism of recurrent disease after LT. The Tfh/Tfr–B-cell circuit nevertheless offers a biologically plausible route to antibody-mediated rejection. In a longitudinal cohort of 55 orthotopic LT recipients, those who developed IRI had higher pretransplant cTfh17 frequencies and donor-reactive IL-17A-producing cTfh responses. These changes accompanied expansion of memory B cells and antibody-secreting cells and the formation of donor-specific antibodies (35). The study is consistent with Tfh–B-cell coordination but does not establish causality or a treatment threshold. Direct evidence for Tfr trajectories after LT is sparse (36, 37).

The CD4^+^ compartment is most informative when represented as interacting trajectories. Serial Th1 activation, Th17/Treg balance, Tfh/Tfr states, B-cell differentiation, and donor-specific antibodies should be assessed alongside drug exposure and graft histology. Such integration could distinguish cellular from humoral immune activation, but current evidence does not support a universal CD4^+^ T-cell threshold for treatment adjustment. **Figure 3** integrates the principal CD4^+^ T-cell pathways and their different levels of clinical support.

**Figure 3:**
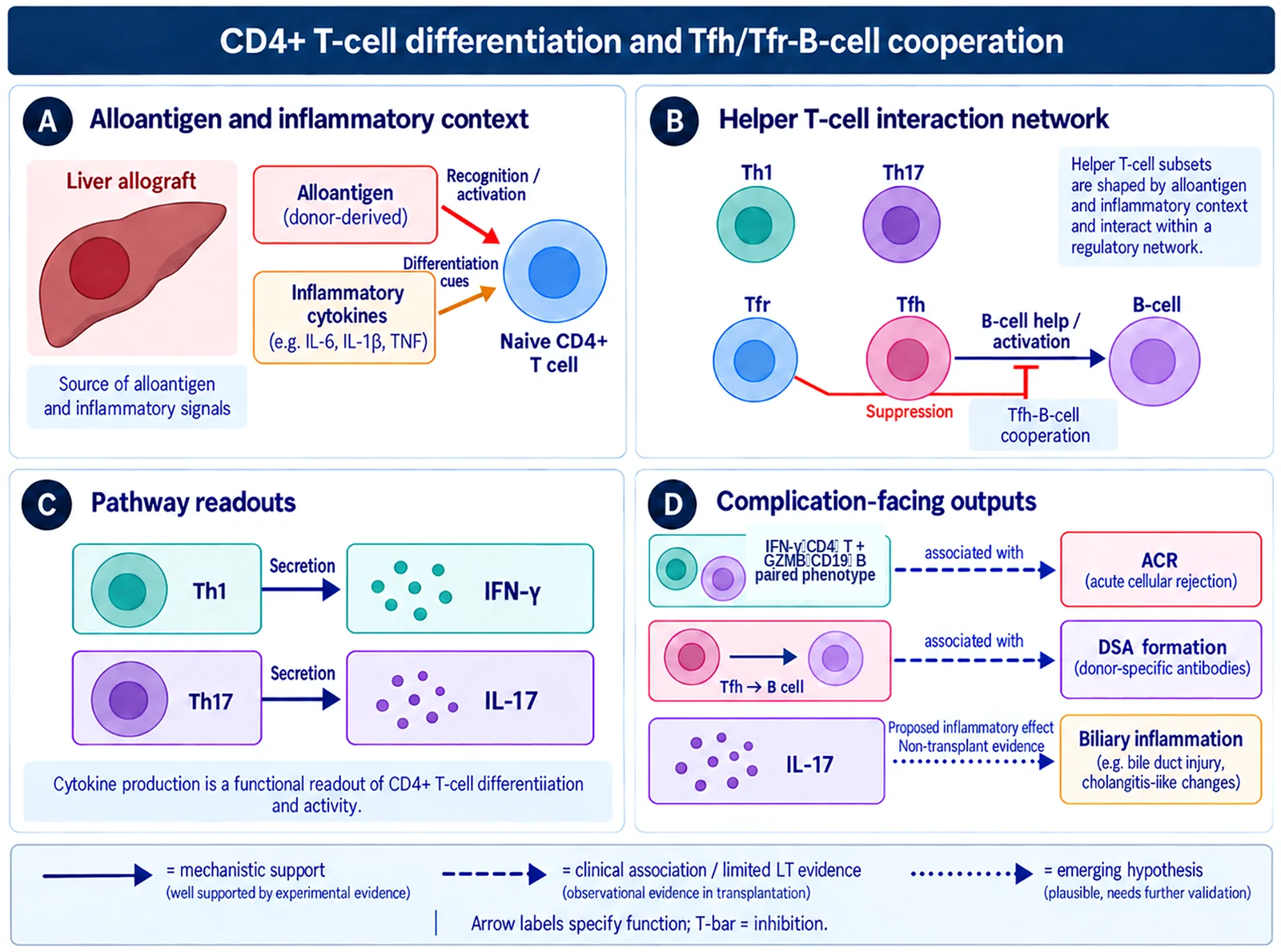
CD4^+^ T-cell differentiation and Tfh/Tfr–B-cell cooperation after liver transplantation. **(A)** Alloantigen recognition and inflammatory cues for CD4^+^ T-cell differentiation. **(B)** Tfh-mediated B-cell help and Tfr-mediated suppression of Tfh–B-cell cooperation. **(C)** Cytokine outputs of Th1 and Th17 cells. **(D)** Clinical associations with acute rejection and donor-specific antibody formation, and a proposed link to biliary inflammation. Th1/interferon-γ activity and combined CD4⁺ T-cell–B-cell effector phenotypes have direct clinical associations with acute rejection. Th17-linked biliary inflammation is supported mainly by non-transplant liver models. Tfh cells provide B-cell help supporting antibody responses, whereas Tfr cells can restrain Tfh–B-cell cooperation. Donor-reactive Tfh responses have been associated with donor-specific antibody formation, while post-transplant Tfr trajectories remain incompletely resolved. ACR, acute cellular rejection; ABMR, antibody-mediated rejection; DSA, donor-specific antibody; Tfh, T follicular helper cell; Tfr, T follicular regulatory cell; Th1, T helper 1 cell; Th17, T helper 17 cell.

### 4.3 Regulatory T cells: functional stability, donor specificity, and graft localisation

Regulatory T cells (Tregs) restrain alloimmune responses through several mechanisms. They consume IL-2, use cytotoxic T-lymphocyte-associated protein 4 (CTLA-4) to modulate CD80/CD86 on antigen-presenting cells, secrete inhibitory mediators, and suppress effector T-cell expansion. Their contribution to graft acceptance depends on FOXP3 stability, suppressive competence, donor specificity, survival, hepatic homing, and interactions with local antigen-presenting cells. FOXP3 expression alone does not establish stable suppression. Outside transplantation, AMP-activated protein kinase α1 (AMPKα1) loss impaired Treg function in models of autoimmune liver disease, whereas Tregs in acute hepatitis A acquired tumour necrosis factor (TNF)-producing, Th17-like features and reduced suppressive activity (38, 39). Hepatocyte-mediated enclysis has also been proposed as a liver-specific regulatory process that could interact with Treg biology (40), but its contribution after LT has not been tested. Treg abundance is an incomplete measure of immune regulation.

Early post-transplant inflammation can weaken this regulatory network. Ischaemia-reperfusion injury and danger signals activate dendritic cells and inflammatory pathways, which can favour effector differentiation and Th17/Treg imbalance. Experimental LT adds a non-linear observation: an initial IFN-γ-associated rejection response can activate mesenchyme-mediated immune control and support later tolerance in mice (41). Other experimental studies implicate high-mobility group box 1 (HMGB1)/nuclear factor-κB (NF-κB) signalling and dendritic-cell activation in rejection-associated disruption of regulatory balance (28, 42). Reviews of regulatory dendritic cells and liver-specific tolerance integrate these pathways but do not establish validated therapeutic targets in human LT (43, 44).

Clinical studies show that Treg kinetics and function are associated with rejection risk. In recipients receiving basiliximab induction and tacrolimus-based immunosuppression, an early decline in circulating Treg frequency was more pronounced among patients with suspected or biopsy-proven acute rejection (29). A separate human study found the lowest proportions of galectin-1-positive Tregs and CD7-positive responder T cells during acute rejection, and CD7 frequency was inversely related to liver-enzyme levels (45). The proposed day-7 Treg threshold came from a single cohort with few biopsy-proven events and cannot be generalized across centres or immunosuppressive protocols. Serial changes in the Treg/Th17 balance have also been associated with tolerance-related phenotypes during immunosuppression tapering (27).

Graft localisation may be as important as peripheral abundance. Operationally tolerant recipients can show enrichment of Tregs within hepatic infiltrates before broad downregulation of proinflammatory genes (46). During immunosuppression withdrawal, extracellular adenosine suppressed human Treg activation, while expression of its degrading enzyme, adenosine deaminase, was higher in tolerant than nontolerant recipients (47). These observations are compatible with local and metabolic regulation, but they do not establish that hepatic Treg enrichment or adenosine handling alone is sufficient to induce operational tolerance.

The low-dose IL-2 trial provides direct evidence against equating circulating expansion with tolerance. Treatment increased peripheral Treg numbers but did not sufficiently expand donor-reactive Tregs, promote intrahepatic enrichment, or permit consistent immunosuppression withdrawal (3). The findings show that numerical expansion must be interpreted together with antigen specificity, trafficking, and graft-level function.

Donor specificity may itself decline after transplantation. Human data indicate a selective reduction in donor-reactive Tregs accompanied by broader activation and senescence-associated changes, which may limit the persistence of donor-specific regulatory therapy (48). Early-phase trials nevertheless show that ex vivo expanded Treg products can be manufactured and administered to LT recipients, and selected protocols have supported immunosuppression reduction under intensive monitoring (49, 50). Reviews of transplant and liver-specific Treg therapy converge on the need to define product identity, antigen specificity, dosing, and compatible immunosuppression (51–56). These studies establish feasibility and biological activity, not routine efficacy. A prospective multicentre immunosuppression-withdrawal study subsequently found biopsy-confirmed operational tolerance in 10 of 61 recipients who initiated withdrawal. Donor-specific T-cell senescence, C4d deposition, and biopsy immune-synapse density emerged as candidate biomarkers but require independent validation (57).

Manufacturing studies provide more specific constraints. good manufacturing practice (GMP)- compatible expansion and cryopreservation can produce functional Tregs from patients with end-stage organ disease, including LT candidates (58). Costimulation-blockade protocols can also expand Tregs from LT recipients, but yield falls when CD4^+^ lymphopenia limits the input population (59). Membrane-bound IL-2 improved chimeric antigen receptor Treg (CAR-Treg) stability and calcineurin-inhibitor resistance in preclinical systems (60). These advances improve product development but do not demonstrate prevention of liver-allograft rejection.

Negative studies also define the limits of translation. In a rat LT rejection model, mesenchymal stromal cells and everolimus each increased Treg levels, but post-transplant stromal-cell infusion neither prevented acute rejection nor acted synergistically with everolimus (61). Treg expansion is best treated as a pharmacodynamic measure rather than a surrogate endpoint for tolerance.

The consequences of Treg activity also vary across clinical outcomes. Stronger regulation may restrain alloimmunity while weakening antimicrobial and antitumour surveillance. CXCL10/CXCR3-associated recruitment of Tregs has been linked to HCC recurrence after LT (62). In endotoxaemia models, hepatic pannexin 1 (PANX1)–IL-33 signalling targeted interleukin-1 receptor-like 1 (ST2)- positive Tregs and accelerated resolution of hyperinflammation (63). This pathway may be relevant to post-LT sepsis, but it has not been validated in septic LT recipients. Studies of Treg-directed interventions need to assess infection and tumour outcomes alongside rejection and tolerance.

A useful Treg monitoring strategy would combine FOXP3 stability, activation and survival markers, suppressive function, donor reactivity, hepatic homing, the Th17/Treg balance, and the surrounding inflammatory state. None has a multicentre-validated threshold for diagnosing tolerance or guiding immunosuppression withdrawal. Prospective studies must link serial blood phenotypes to graft-local regulation and predefined outcomes. **Figure 4** shows why Treg abundance must be interpreted together with specificity, function, and graft localisation.

**Figure 4:**
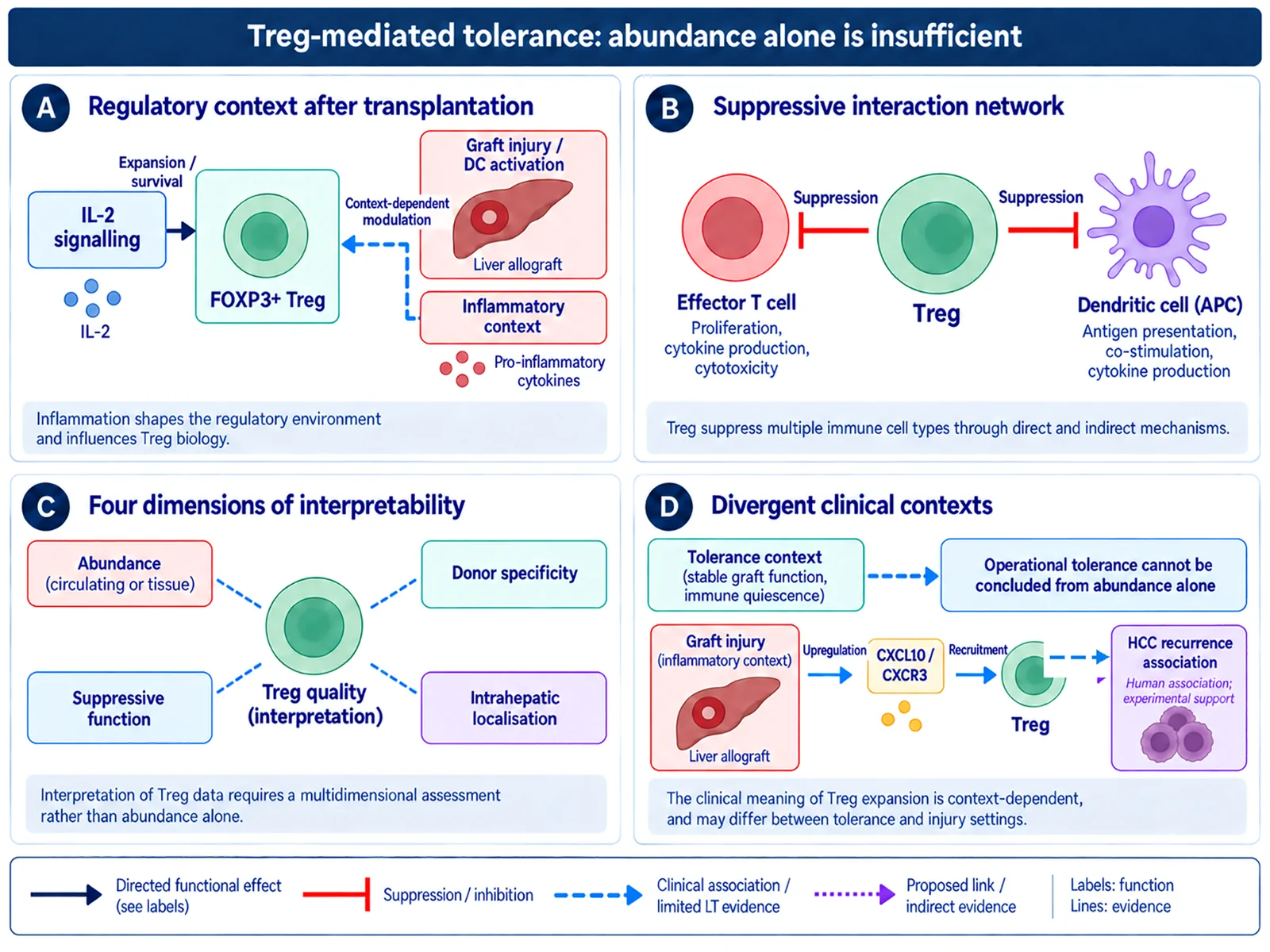
Determinants and context-dependent effects of regulatory T cells after liver transplantation. **(A)** IL-2 signalling and the inflammatory context. **(B)** Treg-mediated suppression of effector T cells and dendritic cells. **(C)** Treg abundance, suppressive function, donor specificity, and intrahepatic localisation. Interleukin-2 availability, graft injury, antigen-presenting cells, donor specificity, suppressive stability, and intrahepatic localisation jointly determine the regulatory trajectory. Increased circulating abundance alone does not establish operational tolerance and may coexist with weak donor reactivity or inadequate graft homing. Tregs suppress effector T-cell responses and dendritic-cell activity, potentially restraining rejection but also weakening antimicrobial or antitumour surveillance. Panel **(D)** depicts CXCL10–CXCR3-associated Treg recruitment and a proposed link to HCC recurrence in an injured graft; this is not an established causal pathway in clinical liver transplantation. CTLA-4, cytotoxic T-lymphocyte-associated protein 4; CXCL10, C-X-C motif chemokine ligand 10; CXCR3, C-X-C motif chemokine receptor 3; HCC, hepatocellular carcinoma; IL, interleukin; Treg, regulatory T cell.

### 4.4 B cells: effector, antibody-producing, and regulatory states

B cells contribute to post-transplant immunity through antigen presentation, cytokine production, antibody generation, and immune regulation. These functions arise from distinct trajectories involving naive and memory B cells, activated effector populations, plasmablasts, plasma cells, and regulatory B cells (Bregs). B-cell abundance alone cannot distinguish protective humoral immunity from alloantibody production or regulatory activity.

Humoral alloimmunity begins when donor-antigen recognition and T-cell help drive B-cell activation, germinal-centre selection, class switching, and differentiation into antibody-secreting cells. Persistent donor-specific antibodies can contribute to complement activation and microvascular injury, culminating in antibody-mediated rejection when serological and histological criteria converge (37). This pathway is well established conceptually, but the temporal relationship between circulating B-cell states, donor-specific antibody formation, and liver-allograft injury remains incompletely defined. Paediatric living-donor LT biopsies and mechanistic experiments further linked tertiary lymphoid structures to rejection and fibrosis through IFN-γ-JAK-STAT-dependent differentiation of atypical memory B cells; JAK1/3 blockade or Lta deletion attenuated rejection in mouse LT models (64).

The most direct cellular evidence for acute rejection concerns a combined peripheral phenotype. Granzyme B^+^CD19^+^ B cells increased together with IFN-γ^+^CD4^+^ T cells in recipients with acute hepatic rejection (1). The association broadens the role of B cells beyond antibody production, but it does not show that granzyme B^+^ B cells independently mediate graft injury or that the combined marker can replace biopsy.

Bregs provide an opposing regulatory programme. Through interleukin-10 (IL-10) and related pathways, they can suppress T-cell activation and inflammatory cytokine production. TIM-1^+^ Bregs have been implicated in experimental regulation of hepatic IRI (6). Evidence for enhanced suppressive function of CD24^hi^CD27^+^ Bregs, however, comes mainly from graft-versus-host disease and other non-liver-transplant settings (65). These observations are compatible with B-cell-mediated regulation but do not define a protective Breg threshold after LT.

B-cell trajectories also influence infection and vaccine responsiveness. Broad suppression of B-cell activation or plasma-cell differentiation may reduce humoral alloimmunity while impairing immunoglobulin production and pathogen-specific memory. Clinical interpretation should include immunoglobulin levels, vaccination history, viral surveillance, and exposure to B-cell-directed or antiproliferative therapy.

Peripheral blood provides only a partial view of humoral immunity. Antibody production can persist in lymphoid tissues and bone marrow despite modest changes in circulating B-cell counts. Paired samples from acute-on-chronic liver failure showed that intrahepatic activated and atypical memory B cells and plasma cells differed from circulating populations (66), illustrating liver compartmentalization outside transplantation. Murine transplantation also shows that donor B cells and other resident lymphocytes can be retained or displaced according to MHC mismatch (16). Serial measurement of donor-specific antibodies, plasmablasts, memory subsets, and B-cell receptor (BCR) clonotypes could clarify whether expanding clones precede antibody-mediated injury or persist after treatment. Such BCR-based trajectories have not yet been validated for routine LT monitoring.

The most defensible clinical approach is context specific. Donor-specific antibody testing remains relevant when graft dysfunction and histology raise concern for antibody-mediated rejection, whereas B-cell phenotyping is still investigational. Future studies should distinguish effector, antibody-producing, and regulatory trajectories and test whether their integration improves discrimination among rejection, infection susceptibility, and stable graft function. **Figure 5** contrasts effector and regulatory B-cell trajectories after LT.

**Figure 5:**
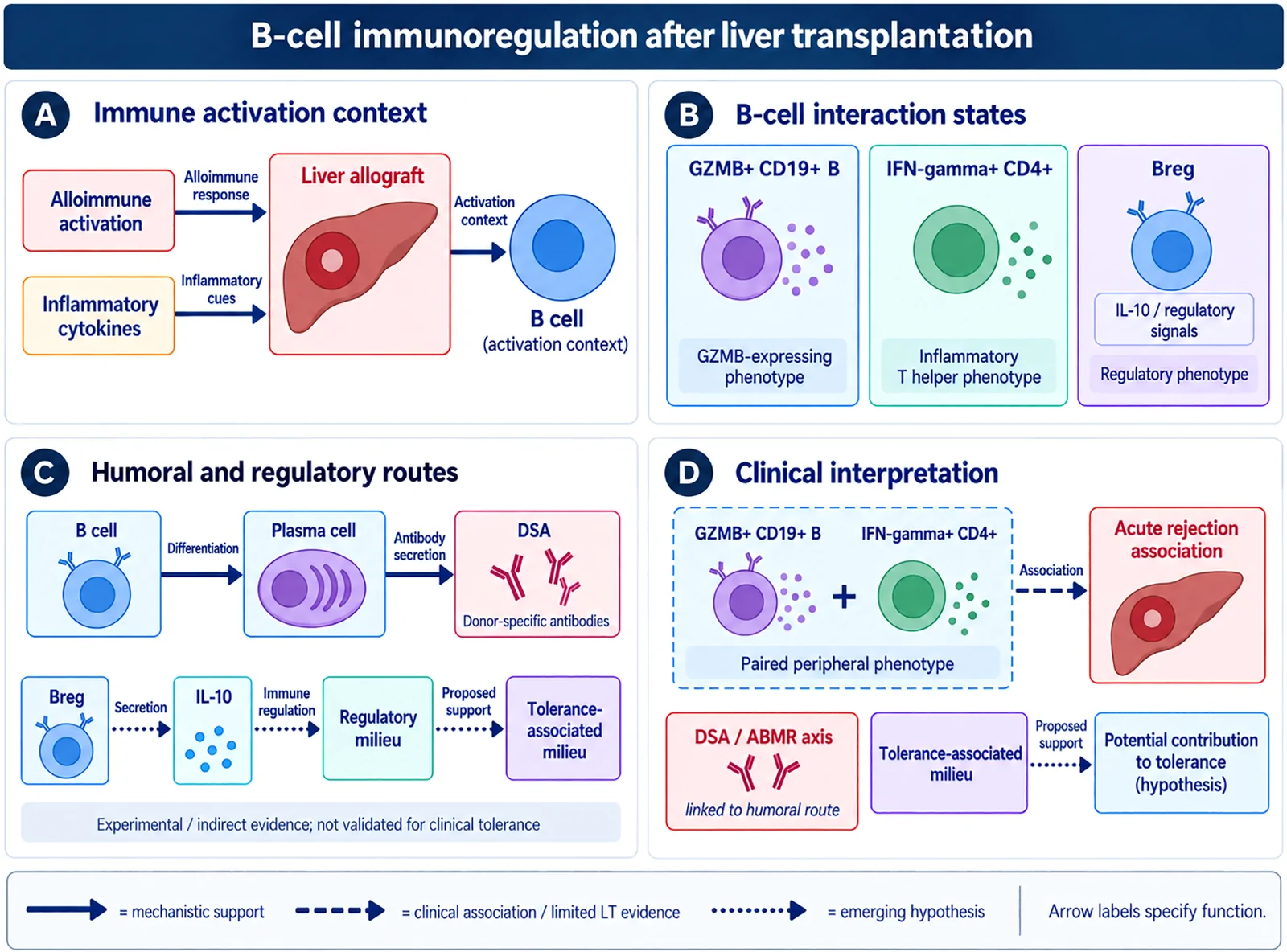
Effector and regulatory B-cell trajectories after liver transplantation. **(A)** Alloimmune and inflammatory contexts for B-cell activation. **(B)** GZMB-expressing CD19^+^ B cells, IFN-γ^+^CD4^+^ T cells, and Bregs. **(C)** Humoral and regulatory routes. **(D)** Rejection-associated phenotypes and proposed regulatory effects. T-cell help can promote B-cell activation and differentiation into antibody-secreting plasma cells, supporting donor-specific antibody production and antibody-mediated injury. A combined granzyme B^+^CD19^+^ B-cell and interferon-γ^+^CD4^+^ T-cell phenotype has been associated with acute rejection; granzyme B expression alone does not establish B-cell cytotoxic function. Regulatory B-cell pathways, including the interleukin-10-associated responses depicted here, have mainly experimental or indirect support, and no validated Breg threshold is available for clinical liver-transplant monitoring. ABMR, antibody-mediated rejection; Breg, regulatory B cell; DSA, donor-specific antibody; GZMB, granzyme B.

### 4.5 Natural killer cells: antiviral surveillance and context-dependent graft injury

Natural killer (NK) cells provide rapid antiviral and antitumour responses but can also amplify graft injury. Their activation reflects the balance between inhibitory receptors, including killer-cell immunoglobulin-like receptors (KIRs), and activating receptors that detect cellular stress or reduced human leucocyte antigen (HLA) class I expression. The post-transplant consequence of an NK-cell response depends on donor-recipient HLA context, inflammatory signals, viral exposure, and antibody availability.

KIR-HLA interactions set the threshold for NK-cell activation, while CD16 enables antibody-dependent cellular cytotoxicity (ADCC). Activated NK cells release IFN-γ, perforin, and granzymes and can target stressed endothelial or parenchymal cells. These mechanisms provide plausible links to alloimmune and antibody-associated injury (14). Direct liver-transplant evidence connecting specific KIR-HLA combinations or NK-mediated ADCC to defined rejection phenotypes remains insufficient for routine risk stratification.

The graft microenvironment can redirect NK-cell function. Ischaemia-reperfusion injury, inflammatory cytokines, and activated endothelium may favour cytotoxicity, whereas immunosuppression and persistent viral stimulation may reduce proliferation or effector competence. The same NK-cell compartment can contribute to tissue injury during inflammatory activation and to graft protection through pathogen and tumour control at other times.

The clearest direct clinical evidence concerns antiviral surveillance. In immunosuppressed children with post-transplant lymphoproliferative disease, CMV and EBV coinfection was associated with a lower frequency of CD56^dim^NKG2A^+^KIR^-^ NK cells and suboptimal EBV control (67). This small paediatric cohort does not establish an adult threshold or prove that loss of this subset caused PTLD. It nevertheless identifies a specific NK-cell state linked to impaired viral control in a defined liver-transplant setting.

NK-cell impairment may also weaken tumour surveillance, whereas excessive activation could intensify inflammatory graft injury. These competing effects make global enhancement or suppression of NK activity difficult to justify. Human transplant studies further show that some donor-derived NK populations are long-lived. Eomes^hi^ liver-resident NK cells have persisted in HLA-mismatched grafts for up to 13 years, whereas donor CD56^dim^ NK and CD56^+^ T-cell populations can be detected transiently in recipient blood early after LT (92, 93). In human liver perfusate experiments, hepatic CD56^bright^ tissue-resident NK cells killed activated allogeneic CD8^+^ T cells *in vitro*, and CD160 engagement enhanced this cytotoxic response, supporting a potential local immunoregulatory role without establishing a clinical tolerance endpoint (94). Importantly, CD3^+^CD56^+^ should be regarded as a phenotypic category rather than a lineage-specific definition of invariant NKT cells, because it may include conventional αβ T cells, iNKT, MAIT, γδ T cells, and other unconventional T-cell states. Clinical studies should distinguish recipient and donor NK-cell origin, maturation state, receptor repertoire, cytotoxic function, and the dominant complication at the time of sampling. In a single-centre retrospective cohort of 134 living-donor LT recipients, pretransplant NK activity of at least 10 pg/mL was independently associated with lower HCC recurrence (hazard ratio 0.27, 95% confidence interval 0.08–0.87) (68). Adjusted overall survival did not reach statistical significance. The threshold requires external validation before clinical use.

Near-term monitoring could combine NK-cell phenotype with viral load and functional assays in recipients at high risk of CMV, EBV, or PTLD. KIR-HLA genotyping and ADCC measurements remain research tools until larger longitudinal studies demonstrate reproducible associations with liver-specific outcomes. Drug exposure and concurrent T-cell dysfunction must be modelled because they can produce similar failures of protective immunity.

NK-cell measurements must be interpreted in clinical context. Preserved cytotoxic competence may contribute to antiviral and antitumour surveillance, whereas activation within an inflamed graft may accompany tissue injury. Current evidence includes plausible mechanisms and selected infection-related associations, but no universal NK-cell signature for rejection, infection, or malignancy. **Figure 6** summarises the balance between NK-cell surveillance and graft injury.

**Figure 6:**
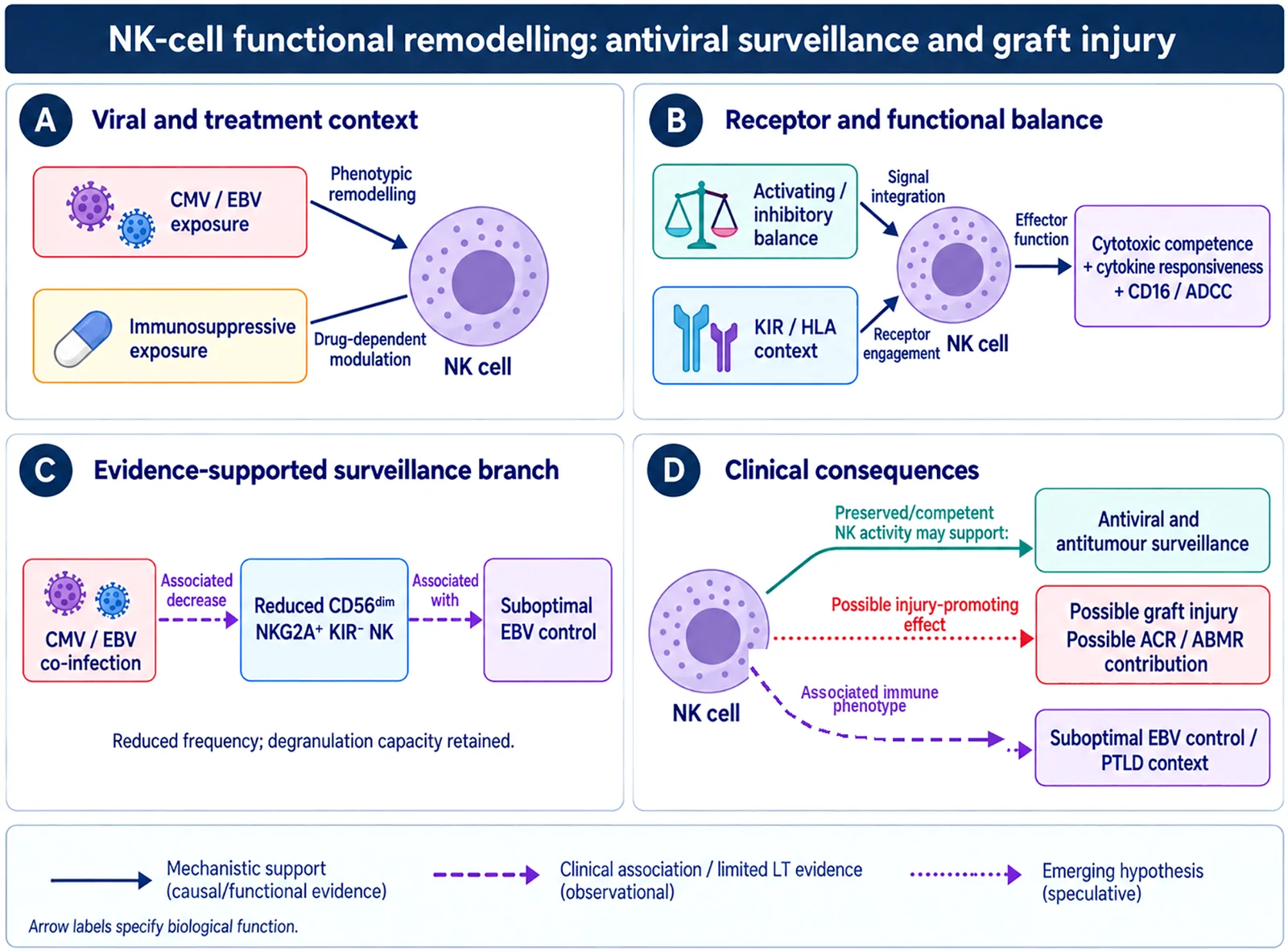
Context-dependent natural killer cell remodelling after liver transplantation. **(A)** Viral and treatment contexts. **(B)** Receptor signalling and functional balance. **(C)** Associations among viral coinfection, reduced frequency of a defined NK-cell subset, and suboptimal EBV control. **(D)** Antiviral and antitumour surveillance, possible graft injury, and impaired viral control. Killer-cell immunoglobulin-like receptor–HLA interactions, antibody-dependent cellular cytotoxicity, viral exposure, and immunosuppression shape NK-cell maturation and function. Preserved cytotoxicity may support antiviral and antitumour surveillance, whereas activation in an inflamed graft could contribute to tissue injury. The surveillance branch includes reduced frequencies of CD56-dim NKG2A⁺KIR⁻ NK cells reported in a small paediatric cohort with post-transplant lymphoproliferative disease and suboptimal Epstein–Barr virus control; this does not establish a validated predictor of future disease. Additional evidence, not explicitly depicted, includes a single-centre adult cohort relating pretransplant NK-cell activity to HCC recurrence. ABMR, antibody-mediated rejection; ADCC, antibody-dependent cellular cytotoxicity; CMV, cytomegalovirus; EBV, Epstein–Barr virus; HCC, hepatocellular carcinoma; HLA, human leucocyte antigen; KIR, killer-cell immunoglobulin-like receptor; NK, natural killer; NKG2A, natural killer group 2 member A; PTLD, post-transplant lymphoproliferative disorder.

### 4.6 Unconventional and innate-like lymphocytes: MAIT, γδ T, iNKT and NKT-like populations

Mucosal-associated invariant T (MAIT) cells, γδ T cells, invariant natural killer T (iNKT) cells, and other heterogeneous NKT/NKT-like populations connect non-classical antigen recognition and cytokine-driven activation with rapid effector responses. The hepatic enrichment of several of these populations places them at the interface of gut-derived signals, sterile injury, biliary inflammation, antimicrobial defence, and repair. Direct evidence linking their longitudinal trajectories to defined post-transplant complications remains limited.

iNKT cells recognise lipid antigens presented by CD1d, whereas other CD1d-reactive and phenotypically defined NKT-like populations are more heterogeneous. A prospective human LT study demonstrated rapid preferential activation of circulating iNKT cells during graft reperfusion and an association with systemic IL-33 release; complementary mouse experiments supported IL-33-dependent recruitment during hepatic ischaemia-reperfusion injury (95). These data provide direct perioperative evidence that iNKT cells participate in acute sterile inflammation, but they do not establish a validated complication threshold or iNKT-directed therapy. More broadly, the distinct antigen-recognition systems of iNKT, MAIT, and γδ T cells raise the possibility of modulating alloreactivity while preserving antimicrobial or antitumour surveillance, but this remains a translational hypothesis rather than an established therapeutic principle in human LT.

MAIT cells recognise microbial riboflavin-pathway metabolites presented by MHC class I-related protein 1 (MR1). They can also respond independently of the TCR to interleukin-12 (IL-12), interleukin-18 (IL-18), and IL-15. Activated cells produce IFN-γ, TNF, and cytotoxic molecules. Tissue studies show functional heterogeneity among human MAIT cells, with liver populations displaying distinct effector characteristics (69). These observations are consistent with a role in the gut–liver axis but do not establish a causal contribution to post-transplant infection or graft injury.

MAIT cells may remain numerically depleted and functionally altered after liver transplantation. Tolerant and conventionally immunosuppressed recipients show different degrees of impairment (5), and longitudinal recovery of CD161^+^Vα7.2^+^ cells has been associated with clinical improvement after transplantation for hepatic failure (70). Recovery in cell number cannot be assumed to restore antimicrobial function.

IL-15 can induce innate-like cytotoxicity in human liver CD8^+^ MAIT cells independently of MR1–TCR signalling (12). Inflammatory cytokines could consequently activate MAIT cells even when microbial antigen presentation is limited. The relevance of this pathway to viral control after LT is unproven, and no MAIT-based intervention or clinical threshold has been validated. **Figure 7** shows the observed and proposed MAIT-cell trajectories after LT. Subsequent single-cell RNA/TCR sequencing and tissue assays identified enrichment of CD8^+^ MAIT cells in human liver allografts and mouse rejection models. MR1 deficiency reduced inflammation and rejection in mice through altered TCR-repertoire remodelling (71). These results provide mechanistic evidence for a rejection-related MAIT programme but do not define a predictive threshold in humans.

**Figure 7:**
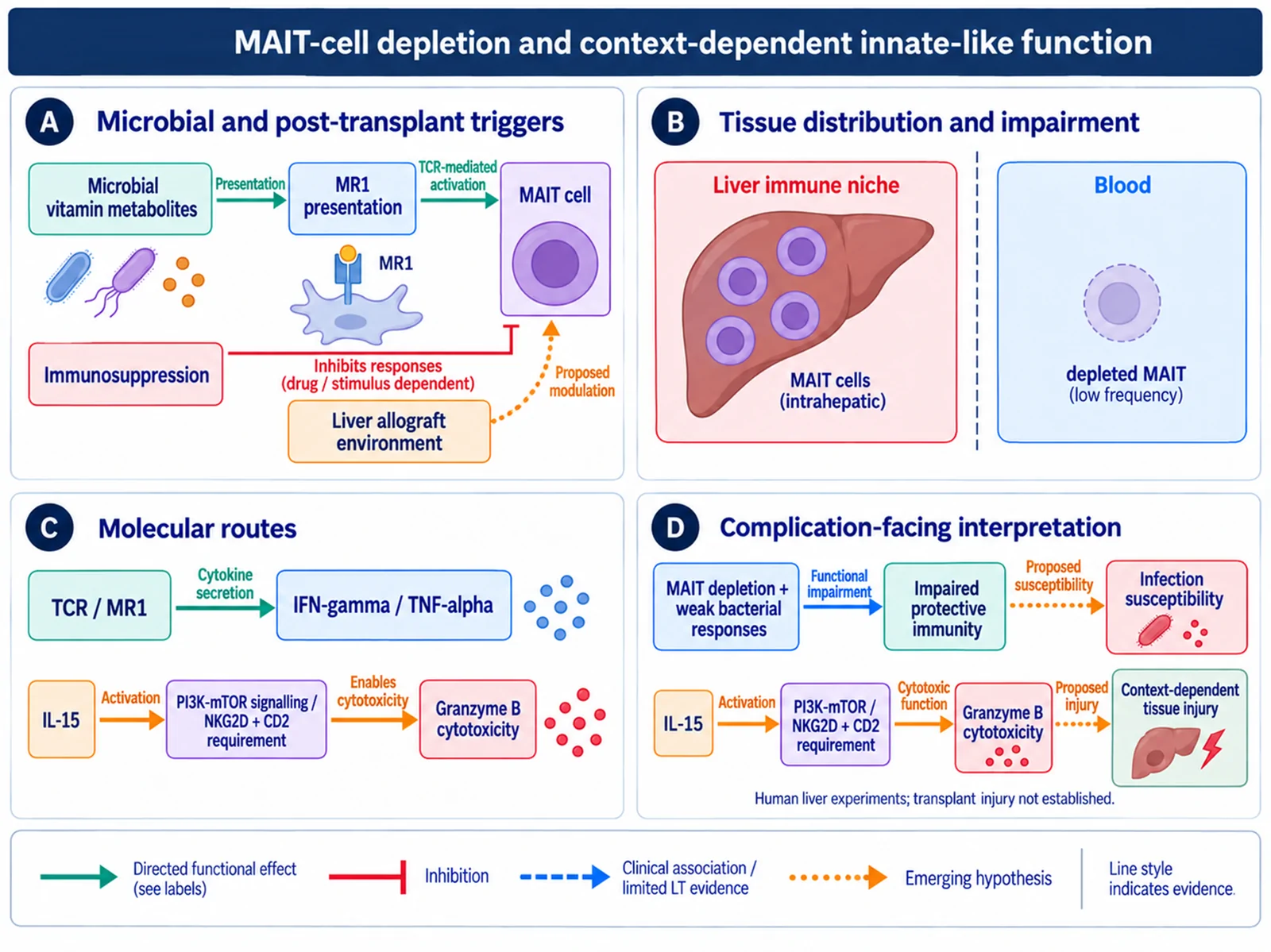
Mucosal-associated invariant T-cell depletion and context-dependent function after liver transplantation. **(A)** Microbial sensing, immunosuppression, and the proposed influence of the graft environment. **(B)** Intrahepatic MAIT cells and reduced circulating MAIT-cell frequency. **(C)** TCR/MR1-mediated cytokine secretion and IL-15-induced cytotoxicity. **(D)** Separate proposed links to infection susceptibility and context-dependent tissue injury. MR1-dependent microbial sensing and cytokine-driven activation can produce antimicrobial or cytotoxic responses. Persistent numerical depletion and impaired bacterial responsiveness have been observed after liver transplantation, while interleukin-15 can induce MR1-independent, granzyme B-dependent cytotoxicity in human liver MAIT cells through mechanisms requiring NKG2D ligation, CD2-mediated conjugate formation, and PI3K–mTOR signalling. The infection-susceptibility and tissue-injury branches represent context-dependent interpretations; the mechanistic findings do not establish causation in human liver-transplant complications, a validated clinical threshold, or a MAIT-directed treatment. IL, interleukin; MAIT, mucosal-associated invariant T; MR1, major histocompatibility complex class I-related protein 1; CD2, cluster of differentiation 2; NKG2D, natural killer group 2 member D; PI3K, phosphoinositide 3-kinase; mTOR, mechanistic target of rapamycin; TCR, T-cell receptor.

Gamma-delta T cells respond rapidly to stress-associated and microbial signals and can produce IL-17, IFN-γ, and mediators involved in tissue repair. Experimental studies have linked IL-17-producing γδ T cells to cholestatic inflammation and bile-duct injury (7, 32). This pathway has been demonstrated in non-transplant models and cannot be directly extrapolated to ischaemic or surgical biliary complications after LT.

Clinical evidence for γδ T-cell therapy after LT is limited to a single case report. Eight allogeneic Vγ9Vδ2 T-cell infusions were administered to an LT recipient with recurrent metastatic cholangiocarcinoma, with no reported treatment-related toxicity and with radiographic and metabolic improvement (72). This observation cannot establish efficacy or general safety in immunosuppressed recipients.

The functional direction of γδ T-cell responses is likely to vary by subset and tissue state. IL-17-dominant responses may amplify neutrophilic inflammation, whereas other populations may support cytotoxic surveillance or epithelial repair. Current liver-transplant datasets do not resolve these functions sufficiently to determine whether γδ T cells are pathogenic, protective, or sequentially both during biliary injury.

Assessment of MAIT and γδ T cells should combine abundance, cytokine production, cytotoxic competence, and tissue localisation. Paired blood, bile, and graft sampling could test whether microbial dysbiosis or biliary injury precedes local recruitment, dysfunction, or depletion. Single-cell and spatial profiling can define these trajectories, but they remain a future research strategy rather than an established mechanism or clinical decision tool. **Figure 8** distinguishes established γδ T-cell biology from unvalidated post-transplant hypotheses.

**Figure 8:**
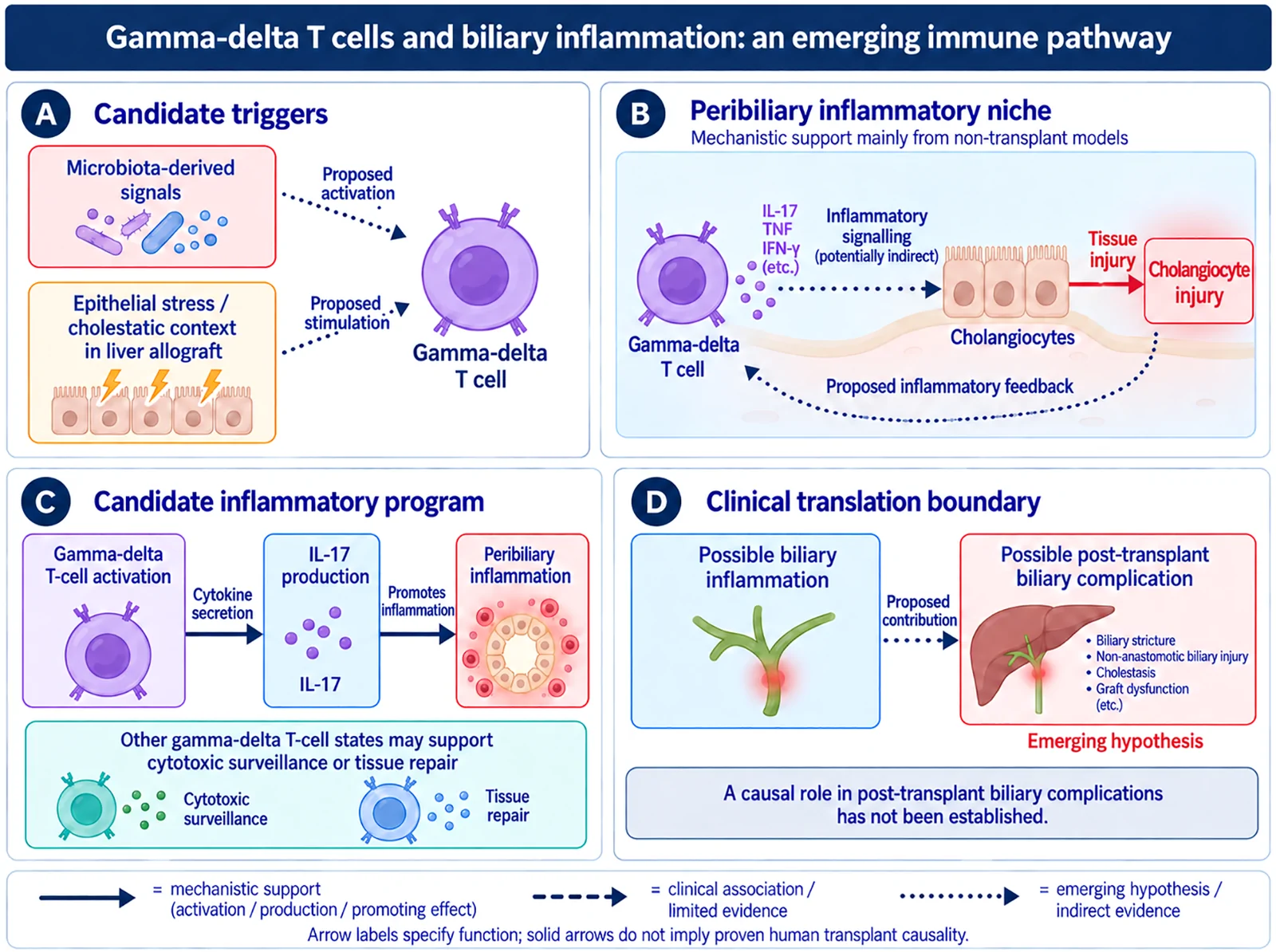
γδ T cells and biliary inflammation after liver transplantation. **(A)** Candidate microbial and epithelial-stress triggers. **(B)** A peribiliary inflammatory niche supported mainly by non-transplant models. **(C)** IL-17-associated inflammation and other candidate γδ T-cell functions. **(D)** Proposed links to post-transplant biliary complications. Microbial and cholestatic signals can activate IL-17-producing γδ T cells and promote peribiliary inflammation in non-transplant models. Other γδ T-cell states may support cytotoxic surveillance or tissue repair. The activation, cytokine-production, and inflammatory effects illustrated here draw mainly on non-transplant models; a causal role in post-transplant biliary complications has not been established. A single case report describes γδ T-cell therapy for recurrent cholangiocarcinoma after liver transplantation, not treatment of post-transplant biliary inflammation. IL, interleukin; TCR, T-cell receptor.

## 5 Complication-level synthesis

The strength of evidence differs markedly among complications. Direct human LT studies most consistently relate lymphocyte measurements to acute rejection, early infection, operational tolerance, selected tumour outcomes, and PTLD. Evidence for biliary, vascular, metabolic, and chronic graft complications is sparse or indirect. This section separates clinical associations from mechanistic extrapolation.

### 5.1 Rejection and tolerance represent opposite outcomes of compartmentalised alloimmunity

Acute cellular rejection has the clearest direct lymphocyte evidence. Peripheral IFN-γ^+^CD4^+^ T cells and GZMB^+^CD19^+^ B cells increase together during rejection, while biopsy studies identify clonally expanded, cytotoxic, tissue-resident, interferon-responsive, and JAK–STAT-associated immune programmes (1, 4, 18–22). Multicellular assessment may be informative, but Banff-classified histology is still required for diagnosis and rejection subtype assignment (37, 73). Outside major inflammatory events, blood and graft TCR repertoires differ sufficiently to limit the use of peripheral measurements as substitutes for tissue assessment (4).

A high circulating Treg count does not establish operational tolerance. Donor-reactive suppressive function, lineage stability, hepatic homing, and a low-inflammatory graft environment distinguish biologically relevant regulation from nonspecific expansion (3, 46–48, 57). Early-phase Treg and regulatory-cell interventions have shown feasibility in selected recipients, but small cohorts, intensive monitoring, and non-randomised designs preclude routine use (49, 50). Rejection and tolerance are better studied through paired blood and graft trajectories than as opposite values of one marker.

### 5.2 Infection risk reflects loss of protective function rather than lymphopenia alone

Pretransplant or early post-transplant PD-1^+^TIM-3^+^ memory T-cell states and reduced IFN-γ production have been associated with early infection (2). Memory phenotypes also vary after viral exposure, but post-infection measurements do not establish prospective risk (74). Results from other cohorts are mixed. Delayed monocyte HLA-DR recovery outperformed lymphocyte measurements in one intensive-care study; first-month CD3^+^ lymphopenia predicted later infection in children; and lower EBV-specific CD4^+^ and CD8^+^ responses accompanied paediatric EBV infection (75–77). The variation across studies argues for functional and pathogen-specific monitoring rather than a universal lymphocyte threshold.

### 5.3 Tumour surveillance and immune regulation create a context-dependent trade-off

Tumour-related evidence is outcome specific. Graft injury can recruit CXCR3^+^ Tregs through CXCL10 and has been associated with HCC recurrence, while lower pretransplant NK-cell activity has also been linked to recurrence (62, 68). Altered NK phenotypes during CMV and EBV co-infection have been reported in paediatric recipients who developed PTLD (67). These studies do not justify a common tumour-risk panel. Regulatory pathways, antiviral competence, viral burden, and tumour-specific clinical risk need to be assessed in parallel.

### 5.4 Evidence for biliary, metabolic, vascular, and chronic complications remains preliminary

IRI has direct human evidence for perioperative CD8^+^ T-cell and circulating Tfh17 trajectories, but links from these signals to later biliary outcomes remain unproven (26, 35). Proposed Th17, Treg, Breg, MAIT-cell, and γδ T-cell pathways in biliary inflammation rely largely on experimental or non-transplant liver studies (5–8, 30–33, 64, 71). Surgical anatomy, ischaemia, infection, and bile toxicity remain major competing explanations. Accordingly, immune-mediated biliary injury should not be inferred from an isolated lymphocyte phenotype.

Current evidence does not define outcome-specific lymphocyte thresholds for metabolic or vascular complications. Chronic graft dysfunction also combines recurrent inflammation, biliary disease, infection, metabolic burden, and drug toxicity. Recurrent autoimmune liver disease can overlap clinically and histologically with rejection, while supporting lymphocyte evidence derives mainly from broader autoimmune-liver cohorts (78–80). Future studies need prespecified complication definitions and serial, paired clinical and immune measurements before causal or predictive claims are warranted.

## 6 Lymphocyte-centred monitoring and translational strategies

Clinical monitoring relies mainly on flow cytometry, sometimes supplemented by TCR-repertoire tracking and *in vitro* functional stimulation. TCR studies show that peripheral blood is an imperfect surrogate for local immunity (4). More informative designs would pair longitudinal blood measurements with graft tissue and spatial transcriptomic or proteomic data. Candidate markers already exist. Pretransplant proportions of PD-1^+^TIM-3^+^ memory T cells may stratify infection risk (2), and combined IFN-γ^+^CD4^+^ T-cell and GZMB^+^CD19^+^ B-cell measurements may identify acute rejection (1). MAIT-cell depletion may indicate impaired mucosal defence during immunosuppression (5). Variable thresholds, assay platforms, treatment exposure, and infection limit their current use. Prospective multicentre calibration is required.

The evidence for intervention varies by setting. Animal models indicate that mesenchymal stromal-cell or cell-engineering approaches can increase Tregs and promote tolerance (81). In human trials, low-dose IL-2 expanded Tregs without establishing tolerance and may have increased intrahepatic inflammation (3). Clinical translation should focus on antigen-specific or trafficking-directed regulation rather than nonspecific expansion. For infection, exhaustion studies favour quantitative assessment of protective immunity when adjusting immunosuppression (2). The CXCL10/CXCR3–Treg axis is relevant to recipients at high risk of tumour recurrence (62). Mechanistic support for intervention is stronger in animal models than consistent clinical evidence in humans. Outcome-oriented longitudinal cohorts with nested mechanistic studies are needed to narrow this gap. The subsections below follow the path from panels and platforms to thresholds, validation, and risk control.

### 6.1 From single markers to interpretable panels: the evolution of monitoring strategies

Clinically interpretable panels combine measurements along an immune axis rather than relying on one cell population. During periods of rejection risk, Th1 or other effector programmes could be paired with B-cell effector phenotypes (1). During periods of infection risk, exhaustion markers are more informative when considered with functional measures such as IFN-γ production (2). Tolerance studies require Treg donor specificity and tissue localisation (3, 11, 48). A feasible panel might include CD4^+^ Th1 and CD8^+^ effector activation, regulatory networks, inhibitory-receptor pathways, and measures of protective immunity. Candidate readouts include Treg proportions and stability markers, PD-1 and TIM-3 expression, and MAIT-cell abundance and function (5). Cellular subsets are highly dynamic and can be altered by sampling time, immunosuppressive exposure, infection or viral reactivation, inflammation, physiological stress, and redistribution between blood and tissue. Soluble immune-mediator or protein signatures can provide a complementary systems-level readout rather than an alternative to cellular monitoring. In the multicentre paediatric ChilSFree cohort, one favourable multivariable plasma soluble-immune-mediator signature emerged by approximately 2 weeks after LT, remained relatively stable during the first post-transplant year, and was associated with rejection-free survival and a higher frequency of CD56^bright^ NK cells (96). The relative stability observed in that cohort should not be generalised to all soluble biomarkers; temporal behaviour remains signature-, population-, and outcome-dependent.

### 6.2 Technology platforms: complementary roles of flow cytometry, CyTOF, single-cell methods, and immune-receptor repertoires

Flow cytometry remains the platform most readily translated into clinical practice because it can support standardised thresholds and longitudinal trends. Cytometry by time of flight (CyTOF) and high-dimensional flow cytometry are useful for discovering subset structures and narrowing them to a small group of informative markers. Single-cell transcriptomics and spatial omics can explain discrepancies between peripheral and intrahepatic immunity by relating cell states to chemotaxis, metabolism, and interactions within the tissue microenvironment. This mechanistic context complements the repeated measurements provided by cytometry. Multiplex plasma protein assays and selected circulating molecular injury markers can be sampled repeatedly with lower tissue burden and may add integrated inflammatory or graft-injury information, but they provide different biological information from cellular phenotyping and should be interpreted jointly rather than treated as substitutes (96, 97). TCR and BCR repertoires add information on clonal expansion, antigenic drivers, and migratory paths; in ACR, the TCR repertoire is particularly informative about spatial compartmentalization (4). Once these platforms are established, the central question becomes how their readouts can guide intervention.

### 6.3 Immunological readouts and risk management for intervention strategies

Evaluation of Treg-directed interventions should centre on donor specificity, intrahepatic homing, and suppressive function, with numerical expansion treated as an incomplete measure. The low-dose IL-2 trial showed that expansion can coincide with adverse inflammatory responses (3). Modulation of pDCs, dendritic cells, or PD-1/PD-L1-related pathways may create an immunosuppressive milieu, but the resulting infection risk must also be considered (2, 9). More broadly, mesenchymal stromal cell therapies and combination regimens may preserve immune balance while reducing drug toxicity. Foxp3-engineered mesenchymal stromal cells, for example, upregulate PD-L1, inhibit T-cell proliferation, and induce a Treg phenotype. Stronger clinical evidence is needed to define the appropriate population, treatment window, and long-term safety (81). Comparison across studies will depend on a common minimum dataset.

### 6.4 Proposed minimum dataset

Greater comparability across cohorts requires standardisation in four domains. The following dataset is intended primarily to standardise prospective translational studies rather than to prescribe routine high-dimensional testing for every LT recipient. Routine monitoring should prioritise readily repeatable clinical, biochemical, pharmacological, virological, and a limited number of prespecified immune measurements, while tissue-based and high-dimensional assays should be reserved for event-triggered assessment or nested mechanistic studies.

- Clinical data should capture longitudinal immunosuppressive exposure, reasons for dose changes, graft biochemistry, CMV or EBV exposure, prophylaxis, and standardised definitions of major complications.
- Peripheral immune data should use a limited prespecified set of cellular and, where appropriate, soluble readouts matched to the study question. Candidate measures include CD4^+^ or Th1 responses with IFN-γ production, CD8^+^ effector and inhibitory-receptor phenotypes, Treg proportions and stability markers, selected B-cell effector phenotypes such as GZMB^+^CD19^+^ cells, MAIT-cell abundance or function, and selected soluble immune signatures undergoing prospective validation (1–5, 96).
- Tissue and mechanistic data should include histopathological classification of biopsies obtained for defined clinical or protocol indications and, where feasible, TCR repertoire or other origin-resolved analyses. These measurements are most appropriate for event-triggered or nested mechanistic studies rather than routine repeated testing.
- Functional data should include at least one prespecified measure of protective or alloreactive immunity when justified by the study aim, such as T-cell IFN-γ production or MAIT-cell responses to bacterial stimulation. These research domains correspond to signals with current translational support, but none constitutes a universal routine clinical panel or validated intervention trigger.

### 6.5 From association to clinical decision-making: thresholds, intervention triggers, and validation pathways

Statistical significance alone is insufficient for clinical use; thresholds and action rules must be specified. Biomarker stability should not be regarded as an intrinsic advantage because the required temporal behaviour depends on the intended clinical use. Diagnostic markers should respond to current immune events; short-term predictive markers must precede outcomes over a defined window; treatment-response markers should change reproducibly with intervention; and long-term risk or tolerance signatures may benefit from greater temporal integration. Cellular, soluble, and molecular readouts may therefore be complementary rather than interchangeable. Age is an important calibration variable because immune markers change markedly in children awaiting LT, requiring age-specific reference ranges for paediatric monitoring (82). Action rules must state whether a functional-exhaustion signal changes antiviral prophylaxis or immunosuppression and whether a T-cell–B-cell signal prompts earlier imaging, biopsy, or treatment adjustment (1, 2). Prospective studies and multicentre validation must then test those rules. Current evidence permits risk stratification and mechanistic inference but does not justify replacement of clinical judgement.

### 6.6 Translational roadmap: from research discovery to routine clinical care

Translation can be considered in three stages. High-dimensional methods first identify informative subsets and states in small cohorts. Mechanistic experiments, including pathway blockade and functional rescue, then distinguish signals with stronger causal support, such as recovery of IFN-γ function after PD-1 or TIM-3 blockade (2). Reproducible signals must next be reduced to compact flow-cytometry panels, with thresholds tested across centres. Prospective strategy trials are required to determine whether panel-guided adjustment of immunosuppression or infection prophylaxis reduces complications and improves clinical outcomes. An associated biomarker cannot become a clinical tool without this final step. Deliberate enhancement of immunity also introduces risks that require separate consideration.

### 6.7 Areas requiring caution: checkpoint pathways and immune enhancement

Checkpoint blockade has changed cancer therapy, but PD-1/PD-L1 and related pathways may help maintain a tolerogenic milieu after transplantation (9) and have also been linked to infection risk (2). Strategies intended to increase antiviral or antitumour immunity require careful selection of recipients and treatment windows, together with monitoring for graft complications. Reversible, short-course, or localised interventions may offer a safer starting point.

## 7 Discussion

### 7.1 A trajectory-based interpretation of post-transplant immunity

Lymphocyte measurements after LT are best interpreted in relation to time, compartment, function, and treatment exposure. Static counts do not reliably distinguish protective immunity, alloimmune injury, adaptive restraint, and immune insufficiency. In acute rejection, peripheral multicellular signals may identify an activated state, whereas graft clonotypes and tissue programmes define the local process (1, 4, 18–22). Infection and tolerance require different readouts. Inhibitory-receptor expression is more informative when accompanied by functional loss; circulating Treg expansion requires evidence of donor specificity, suppressive stability, and graft localisation (2, 3, 46–48, 57).

Many inconsistencies across studies can be traced to timing, compartment, functional state, or treatment exposure. A perioperative marker may reflect IRI, whereas the same marker months later may reflect persistent antigen exposure, viral reactivation, or drug effects. Peripheral blood permits serial monitoring but does not capture all graft-resident states, and graft-local phenotypes can represent a mixture of persistent donor-derived cells and recipient-derived cells that acquire tissue-resident features (91–94). Functional assays distinguish abundance from competence, although interlaboratory variability limits comparison. Soluble immune-mediator signatures and selected graft-injury markers can add complementary temporal information, but they should not be interpreted as replacements for cellular or tissue-based measurements (96, 97). **Figure 1** summarises this approach; it is not a numerical model.

### 7.2 Current controversies

Checkpoint expression illustrates one current controversy. PD-1 and TIM-3 can mark dysfunctional protective immunity associated with infection, yet inhibitory pathways may also restrain alloimmune injury (2, 9). Their clinical meaning varies with cell type, antigen context, timing, and functional capacity. Treg expansion presents a related problem: a measurable increase in peripheral cells may support tolerance or simply accompany immunosuppression, depending on donor specificity and intrahepatic localisation (3, 46–48). Blood-based prediction is also limited because agreement between blood and graft repertoires increases mainly during strong inflammatory events (4). No single direction of effect can be assigned to checkpoint molecules, Tregs, or other lymphocyte populations across all clinical settings.

A further controversy concerns causal attribution in biliary and chronic complications. Immune pathways are biologically plausible, but surgical, vascular, infectious, and toxic causes often coexist. Experimental IL-17, MAIT-cell, γδ T-cell, or Breg findings cannot establish an immune mechanism in a clinical biliary event without compatible histology, timing, and exclusion of competing causes (5–8, 30–33). Similar caution applies to tumour outcomes, where regulatory immune states may reflect graft injury, cancer biology, treatment, or their interaction.

### 7.3 Limitations of the evidence base and this review

Most clinical studies include small cohorts, cross-sectional sampling, heterogeneous phenotype definitions, and inconsistent endpoints. Immunosuppressive exposure creates confounding by indication, while age, viral history, prophylaxis, graft quality, and transplant indication add heterogeneity. Reverse causation remains difficult to exclude because infection, rejection, and their treatment can each remodel immune phenotypes. Few studies provide external validation or prespecified thresholds.

Peripheral sampling introduces spatial bias, whereas clinically indicated biopsy introduces selection and timing bias. Studies rarely synchronise blood phenotyping, drug levels, virology, and Banff-classified tissue measurements (4, 37, 73). This Review also has methodological limitations. The initial evidence corpus did not arise from one registered search, and heterogeneous study designs precluded formal risk-of-bias grading or meta-analysis. A structured narrative synthesis can compare mechanisms and evidence boundaries but cannot establish completeness or pooled certainty.

### 7.4 Paediatric and adult liver transplantation: developmental context and limits of cross-population extrapolation

The same broad interpretive principles—time, anatomical compartment, cellular function, treatment exposure, and clinical context—are relevant to paediatric and adult LT, but absolute values and trajectories are not directly transferable between these populations. Immune maturation changes naive and memory lymphocyte composition, age-related reference ranges, prior antigen and viral exposure, and the kinetics of immune reconstitution. Paediatric and adult cohorts may also differ in common transplant indications, graft–recipient relationships, CMV/EBV exposure history, and immunosuppressive practice. Prospective paediatric studies show that age-adjusted longitudinal cellular monitoring can identify infection-associated immune deficits, while multicentre plasma profiling has identified early rejection-free signatures with an accompanying CD56^bright^ NK-cell phenotype (76, 82, 96). These findings support shared biological principles but not shared numerical thresholds. Paediatric cellular, soluble, and functional signatures therefore require age-specific calibration, and adult findings should likewise not be transferred to children without direct validation.

### 7.5 Requirements for clinical translation

Translation requires sequential validation. High-dimensional discovery studies may identify informative states, after which reproducible signals must be reduced to compact assays with prespecified sampling windows and thresholds. These assays require validation across centres and clinical eras. Strategy trials must then determine whether marker-guided management improves graft outcomes, infection control, or safe reduction of immunosuppression. Harms such as opportunistic infection, tumour recurrence, unnecessary biopsy, and excessive immunosuppression must be measured at the same time.

Outcome-specific panels are more defensible than a universal immune score. Rejection panels should integrate effector T-cell activity, selected B-cell phenotypes, donor-specific antibodies, and histology. Infection panels should combine inhibitory receptors with functional or pathogen-specific assays and viral burden. Tolerance studies should prioritise donor-reactive Tregs, lineage stability, hepatic homing, and graft inflammation. Each panel requires age-appropriate reference ranges, assay harmonisation, and prospective calibration before clinical use.

### 7.6 Artificial intelligence and multi-omic integration

Artificial intelligence can integrate serial clinical, pharmacological, pathological, and immune measurements, but published LT models rarely include longitudinal lymphocyte phenotypes. Existing studies have predicted graft failure, rejection, sepsis, tacrolimus exposure, or histological regions from clinical, laboratory, molecular, physiological, pharmacokinetic, or imaging data (83–90). Performance within development datasets does not establish a lymphocyte mechanism or clinical benefit.

An immune-trajectory model would require prospective temporal data, recipient-level separation of training and evaluation sets, and external validation across centres. Evaluation should report discrimination, calibration, sensitivity to missing data, subgroup performance, and performance drift. Feature importance and attention weights describe how a model uses variables; they do not establish causality. Artificial intelligence remains a research method until a prospective strategy trial demonstrates net clinical benefit.

### 7.7 Research priorities

Prospective cohorts should collect a common minimum dataset covering sampling windows, immunosuppressive exposure, viral status, lymphocyte phenotypes, functional assays, biopsy classification, and complication endpoints. Paired blood and graft sampling is needed to determine which circulating changes reflect local immune programmes. Studies should define whether the intended use is diagnosis, short-term prediction, treatment-response monitoring, or long-term risk estimation because each purpose requires a different design and threshold.

Mechanistic studies should prioritise reproducible signals that precede clinical events and retain biological specificity after adjustment for treatment exposure. Intervention studies can then test whether modifying those signals improves outcomes without compromising antimicrobial or antitumour immunity. Until these requirements are met, lymphocyte trajectories are suitable for mechanistic interpretation and research stratification, not routine treatment selection.

## 8 Conclusion

Lymphocyte phenotypes after LT change over time, differ between blood and graft, and have outcome-specific meanings. Acute rejection has the strongest direct evidence, combining peripheral effector T-cell–B-cell signals with graft-local clonal and tissue programmes. Early infection is more closely related to impaired protective function than to inhibitory-receptor expression or lymphocyte counts alone. Circulating Treg abundance cannot establish operational tolerance without donor specificity, suppressive stability, and evidence from the graft. Cellular origin and tissue residency provide an additional interpretive layer within the allograft (91–94), while soluble immune signatures and selected circulating graft-injury markers can provide complementary, non-equivalent information (96, 97).

Studies of tumour recurrence and PTLD have identified regulatory, NK-cell, and antiviral associations, but the findings are cohort and outcome specific. Proposed lymphocyte mechanisms for biliary, vascular, metabolic, and chronic complications require more direct clinical evidence. Experimental or indirect pathways should remain hypotheses until prospective human LT studies establish temporal and outcome-specific relationships.

The priority is coordinated validation, not expansion of isolated marker lists. This will require standardised longitudinal sampling, paired blood and graft assessment, functional assays, explicit clinical endpoints, external validation, and strategy trials. Such studies can determine whether lymphocyte trajectories add actionable information beyond clinical variables, drug exposure, virology, and histology.
